# 2-Oxindole and related heterocycles: synthetic methodologies for their natural products and related derivatives

**DOI:** 10.1039/d3ra02217j

**Published:** 2023-05-11

**Authors:** Shivangi Sharma, Yukti Monga, Ashu Gupta, Shivendra Singh

**Affiliations:** a Department of Applied Chemistry, Amity School of Engineering and Technology, Amity University Madhya Pradesh Gwalior Madhya Pradesh-474 005 India shivendrasngh0@gmail.com; b Shyamlal College, Department of Chemistry, University of Delhi Delhi-110032 India

## Abstract

Natural goods, medications, and pharmaceutically active substances all contain substituted oxindoles. Generally, the C-3 stereocenter of the substituents of oxindoles and their absolute arrangement have a substantial impact on the bioactivity of these substances. In this case, the desire for contemporary probe and drug-discovery programs for the synthesis of chiral compounds using desirable scaffolds with high structural diversity further drives research in this field. Also, the new synthetic techniques are generally simple to apply for the synthesis of other similar scaffolds. Herein, we review the distinct approaches for the synthesis of diverse useful oxindole scaffolds. Specifically, the research findings on the naturally existing 2-oxindole core and a variety of synthetic compounds having a 2-oxindole core are discussed. We present an overview of the construction of oxindole-based synthetic and natural products. In addition, the chemical reactivity of 2-oxindole and its related derivatives in the presence of chiral and achiral catalysts are thoroughly discussed. The data compiled herein provides broad information related to the bioactive product design, development, and applications of 2-oxindoles and the reported techniques will be helpful for the investigation of novel reactions in the future.

## Abstract 2

2-Oxindole based natural products and bioactive molecules.

## Graphical abstract

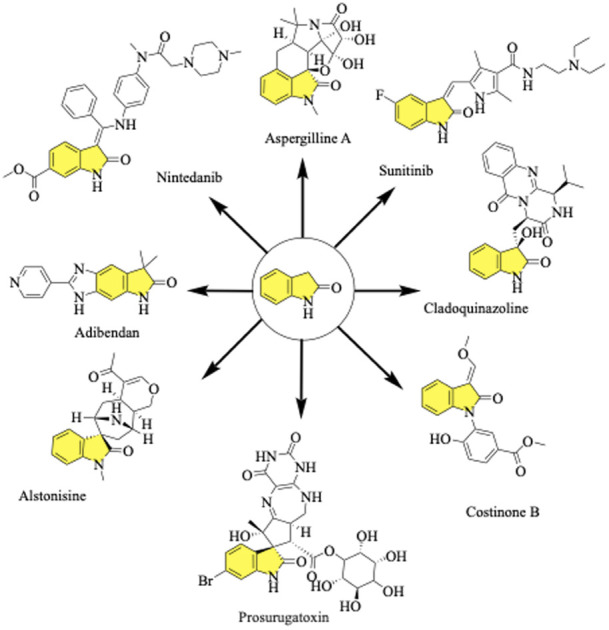

## 1. Introduction

**Fig. 1:**
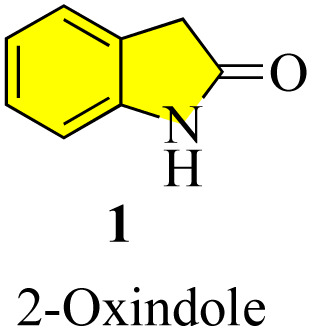
Structure of oxindole.

Oxindoles (1, Fig. 1) are a group of endogenous hetero-aromatic organic compounds (quinoline,^1–3^ indole,^4^*etc.*) found in the tissues and bodily fluids of mammals and in the natural products in different plants.^5^ The term “oxindole” and its derivatives are known as “1,3-dihydro-2*H*-indole-2-one(s)”^6^ because their structure consists of a six-membered benzene ring fused with a five-membered pyrrole ring and a carbonyl group at the C-2 position. It is well known that oxindoles have two rapidly tautomerizing hydroxyl isomers.^7–9^ Several synthetic methods have been successfully applied to develop various derivatives and scaffolds with a range of biological activities, in addition to natural methods to obtain the oxindole nucleus.

**Fig. 2:**
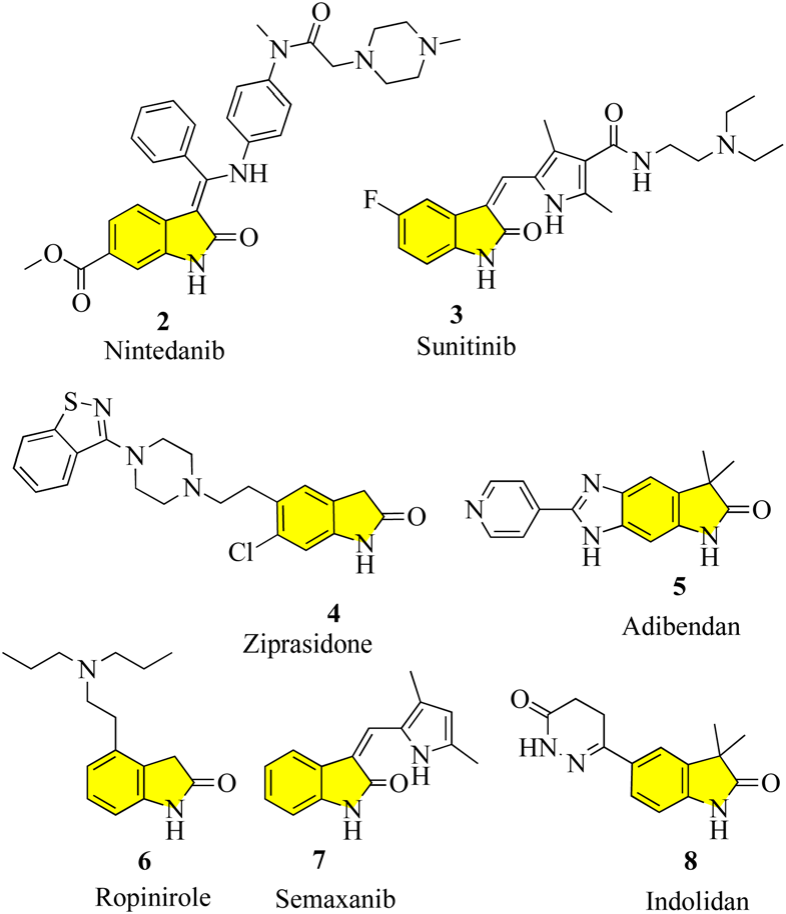
Structure of commercially available drugs with an oxindole core.

New oxindole compounds with a distinct pharmacological profile and commendable efficacy are of great interest in many sectors of the pharmaceutical industry and academia. Nintedanib (2), a marketed highly potent drug, was recently approved in March 2020 in the United States for the treatment of interstitial lung diseases such as idiopathic pulmonary fibrosis (IPF) and chronic fibrosis with a progressive phenotype. It is one of the most potent indolinone compounds and has an effective antiproliferative property, which inhibits angiokinase and limits the growth factor. Nintedanib is advertised commercially under the brand names “Ofev” and “Vargatef”. ^10^ Sunitinib (3), a tiny molecule that primarily serves as a tyrosine kinase inhibitor and a well-known treatment for renal cell carcinoma and gastrointestinal stromal tumours, is another notable oxindole derivative. It was clinically approved by the Food and Drug Administration (FDA) in 2006 and is the first anticancer medicine to be approved for use on two distinct types of cancer cell lines simultaneously. As part of its mechanism of action, it indirectly targets numerous receptor tyrosine kinases in an effort to suppress cellular signaling.^11^ The phase III trials for semaxanib (7), a tyrosine kinase inhibitor that targets angiogenesis and colon-rectal cancer *via* the vascular endothelial growth factor pathway, were unsuccessful.^12^ Ropinirole (6), a commonly prescribed drug for the treatment of Parkinson's disease and restless legs syndrome (RLS), is a well-known medication, which contains the widely used active ingredient oxindole. It was developed in 1996 and functions as a complete agonist at D_2_, D_3_, and D_5_ receptors, as well as a dopamine receptor (D_2_) agonist. However, it has a relatively lower affinity for D_1_ and D_5_ receptors. The success of ropinirole as a medication is attributed to the characteristics of its structure, which are beneficial to its functionality, such as low molecular weight, accessibility, and a stereocenter-free chemical structure, attracting additional attention in ongoing research.^13^ Another oxindole derivative called ziprasidone (4) is a novel antipsychotic medication produced by the American pharmaceutical behemoth Pfizer and marketed and sold under the trade name “Geodon”. Presently, ziprasidone has received FDA approval for use in psychotherapy and the treatment of mental illnesses including schizophrenia and severe manic behavior. Ziprasidone (4) works similarly to ropinirole by blocking the dopamine (D2) receptor.^14^ Cardiovascular diseases and ischemic chest pains are actively treated with some other drugs that have potent vasodilatory and advantageous inotropic effects, notably adibendan (5) and indolidan (8) (Fig. 2).^15^

## 2. Natural products & biologically active compounds

**Fig. 3:**
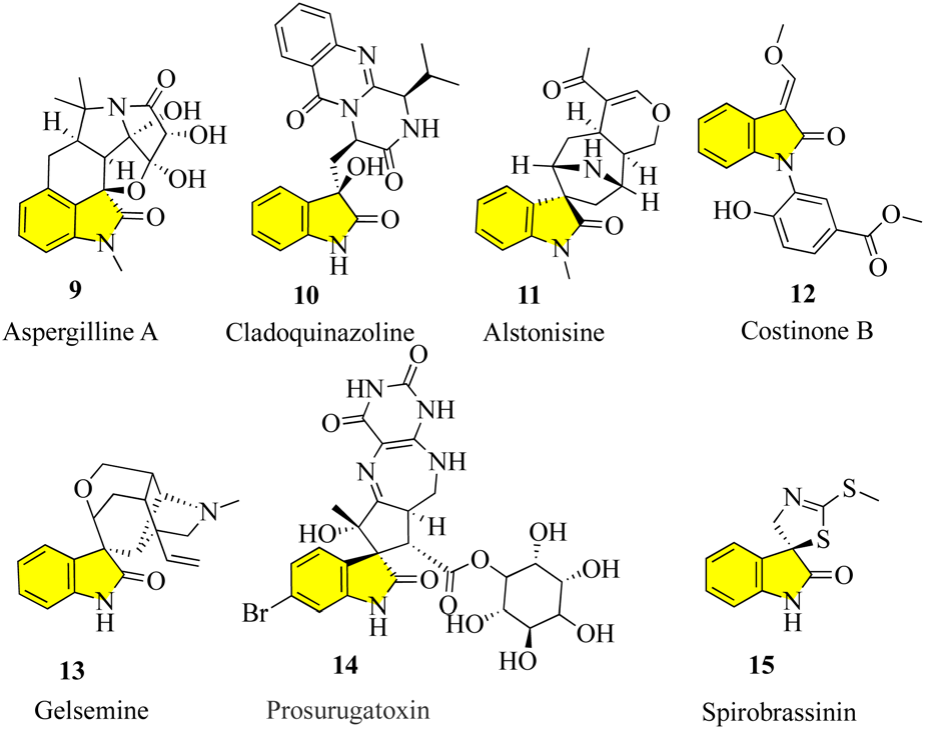
Structure of natural-based bioactive natural products.

To maximize the achievements in current probe- and drug-discovery studies, there is a great demand for synthetic libraries of chiral molecules that mimic the structural characteristics of privileged scaffolds frequently occurring in natural products and pharmaceuticals. One of these classes of scaffolds are 3,3-disubstituted oxindoles,^16^ which are the foundation of many bioactive natural products (Fig. 3). Many new drugs and lead compounds have been created by taking inspiration from these molecules. It is interesting to note that in all fully substituted stereocenters, whether spiro or not, all their carbons are either quaternary or tetrasubstituted with heteroatoms, including the C-3 carbon of oxindoles.^17,18^ The efficient construction of tetrasubstituted, and in particular, quaternary carbon stereocenters remains challenging, which has sparked intense interest in the catalytic enantioselective synthesis of 3,3-disubstituted oxindoles.^19–22^ In addition to providing libraries of structurally different oxindole derivatives for medicinal study, which aid in the discovery of more potent and selective analogues, enormous research effort has also been devoted to the development of new synthetic methodologies.^23^

Notably, successful catalytic enantioselective reactions involving direct C–H bond^24^ functionalization or highly stereoselective construction of adjacent all-carbon quaternary stereocenters are well documented.^25^ A comprehensive overview on the development of the rapidly evolving field of catalytic enantioselective synthesis of 3,3-disubstituted oxindoles was reported in 2010.^26,27^ Meanwhile, it was noted that, although elegant protocols are available, it is still highly desirable to create effective techniques for extracting oxindoles with a wide range of structural compositions from readily available starting materials. To achieve the catalytic^28^ enantioselective synthesis of 3,3-disubstituted oxindoles, some programmes employ new chiral catalysts, new activation models, and tandem sequences.^29^

Aspergilline A (9) was isolated in 2014 from the fungus *Aspergillus versicolor* by Hu and Gao.^30^ Cladoquinazoline (10) is an active obtained from a mangrove-derived fungal strain known as *Cladosporium* sp. and was chosen for additional study as part of anti-influenza compounds. The chemical analysis of the EtOAc extracts of the mycelia of the fungus and its fermentation broth led to the discovery of new indole alkaloids, including cladoquinazoline and other indole alkaloids known to contain quinazoline.^31,32^ Alstonisine (11) was discovered in 1972 by Elderfield and Gilman, which is the first oxindole alkaloid related to macrolines, in the plant *Alstonia muelleriana* Domin.^33,34^

Costinone B (12) was isolated from the Pakistani herb *Isatis costata* and found to inhibit lipoxygenases and butyl cholinesterases. The *N*-aryl substitution in these compounds is notable, and also the presence of the 3-oxygen substituent in costinone B. The presence of the 3′-oxygen substituents in isatinones is noteworthy.^35^

Gelsemine (13, C_20_H_22_N_2_O_2_) is an indole alkaloid that acts as a paralytic and is extremely toxic, which was discovered in flowering plants of the genus *Gelsemium*, a plant native to the subtropical and tropical Americas and Southeast Asia. Exposure to gelsemine can be fatal. The family Loganiaceae, which includes the subtropical to tropical flowering plant genus *Gelsemium*, contains five species as of 2014, with the species *G. sempervirens* Ait. being more common in the Americas and *G. elegans* Benth. in China and East Asia.^36,37^

The chemistry of marine bacteria has also been studied, but they represent a largely untapped source of unusual, bioactive chemical components. Prosurugatoxin (14) and a few brominated pyrroles are examples of products of marine bacterial metabolism that have been isolated thus far, demonstrating that the few genera under investigation appear to utilize a variety of secondary metabolic pathways.^38^

Spirobrassinin (15) belongs to the class of organic compounds known as indolines including (*S*)-spirobrassinin. Indolines are substances that contain an indole moiety, which is created when a pyrrolidine ring is fused to a benzene ring to develop 2,3-dihydroindole. In different foods, including *Brassica oleracea* var. *botrytis*, *Brassica rapa*, and *Raphanus sativus*, spirobrassinin has been identified but not quantified. Consequently, (*S*)-spirobrassinin may one day serve as a biomarker for consuming these foods.^39^

## 3. Pharmaceuticals containing the 2-oxindole moiety

**Fig. 4:**
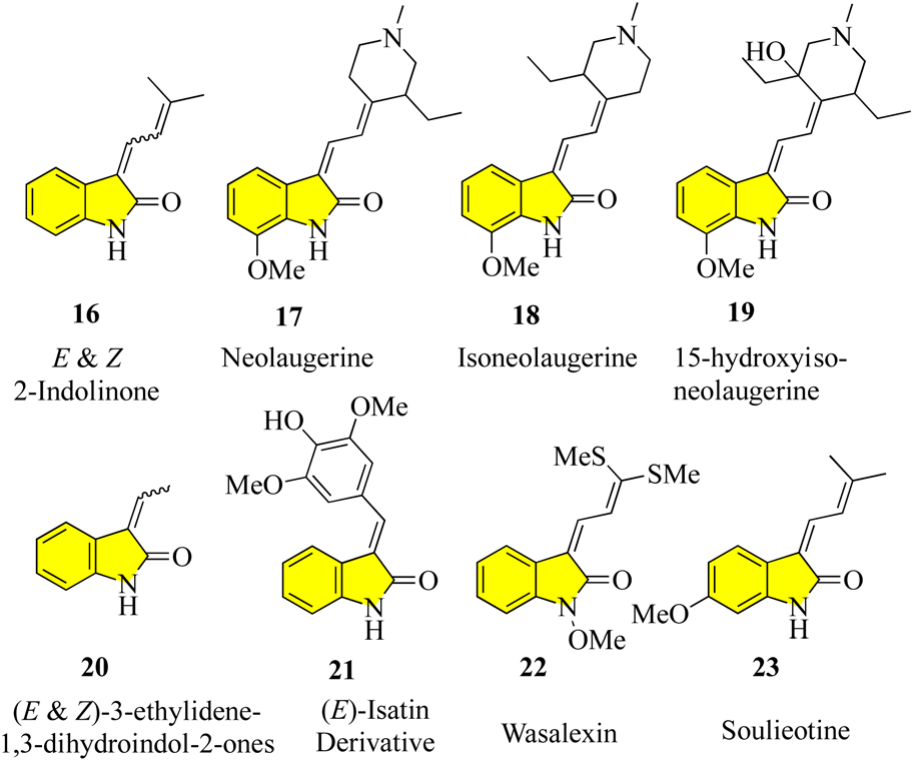
Natural products containing 3 alkyl-oxindole cores and 3 alkyl-tri-substituents.

**Fig. 5:**
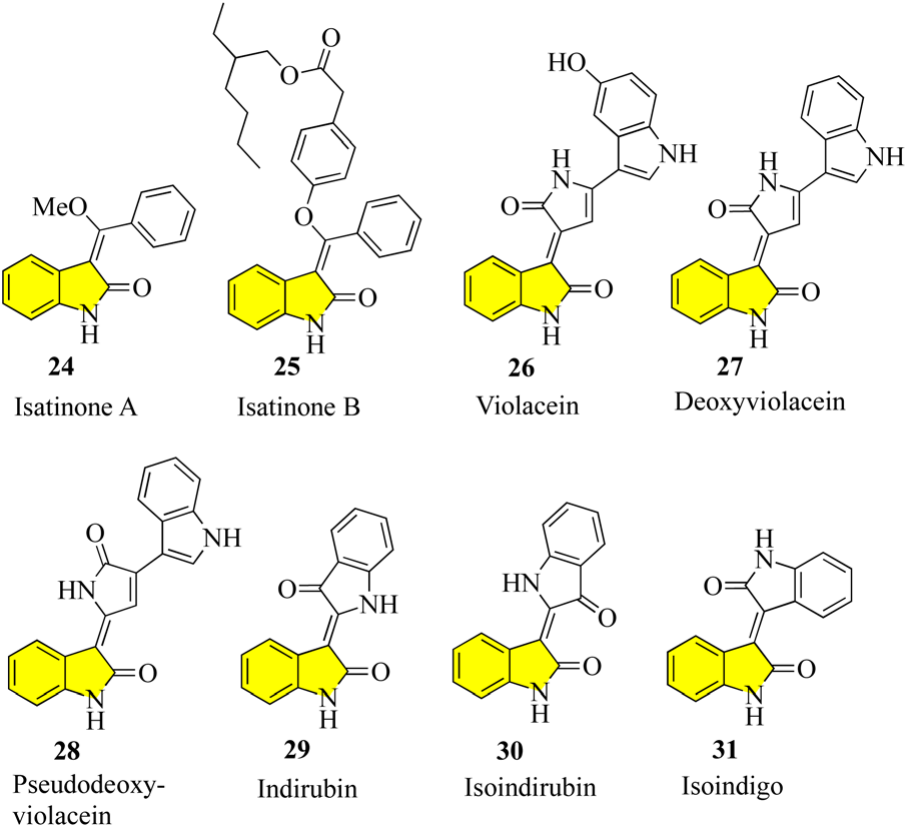
Natural products with a 3-alkenyl-oxindole core and 3-alkene tetra-substituent.

Despite the large number of 3′-alkyl-oxindoles reported, a Beilstein database search, which was limited to structures from natural sources, yielded only 13 compounds with a tri-substituted unit (Fig. 4). The first naturally occurring 3′-alkyloxindoles were (*E*) and (*Z*)-3-(2-methyl-2-butene)-2-indolinones 16 of type 2. In 1978, the two yellow pigments were isolated from the stem of the plant *Cimicifuga dahurica*, which is used in traditional Chinese medicine (Bei Sheng Ma), and especially known for its antipyretic properties. However, it should be noted that the complete description of the structure of these compounds was only reported in 1981 by the same authors. In 1993, three new oxindole alkaloids, *i.e.*, neolaugerine (17), isoneolaugerine (18), and 15-hydroxyisoneolaugerine (19) (no stereo morphisms were fully understood), were isolated from the root of the small evergreen tree *Neolaugeria resinosa*, which is distributed in the Bahamas and West Indies. In 1996, very simple (*E*)-3-ethylidene-1,3-dihydroindol-2-ones 20 were isolated from the *Colletotrichum fragariae* fungus and described as self-growth inhibitors. Oxide alkaloid *E*-isatin derivative 21 was isolated in 1997 from the roots of *Isatis indigotica*, a component of the widely used traditional Chinese medicine Ban-Lan-Gen. In 1999, two plant alkaloids, wasalexins, were isolated from the foliar tissue of Wasabi (*Wasabia japonica*, syn. *Eutrema wasabi*) and wasalexin 22 exhibits antifungal activity against *Phoma lingam*. The functions of the C-3-nitrogen substitute and *N*-methoxy-oxindole are remarkable in these examples. In 2005, it was reported that soulieotine (23) was isolated from the roots of *Souliea vaginata*, a plant used as an anti-inflammatory analgesic in traditional Chinese medicine.^40–47^ The simplest examples (without additional rings) are the two new antifungal alkaloid isatinones A (24) and B (25) isolated from *Isatis costata* (non-stereochemistry defined in the alcohol side chain) in 2007, which expanded the structure search to include naturally occurring 3′-alkenyl-oxide compounds with a 3′-alkenyl unit substituted by tetrasubstituted 3′-alkenyl-oxide compounds, producing the compounds shown in Fig. 5. The presence of a substitute of three oxides in isatinones is noteworthy (see costinone B (12)). There are a sufficient number (six) of natural products of 3′-alkenyloxindole, and the 3′-alkenyl substitute is part of an additional ring. The purple pigment violacein (26) was first isolated from the Amazon bacteria *Chromobacterium violaceum* 1934, has a variety of biological activities, including *in vitro* antitumor effects. The main component of deoxyviolacein (27) was isolated from the same source in 1958, and pseudodeoxyviolacein (28) was isolated from *Chromobacterium violaceum* in 1994. Biosynthetic studies of violacein and deoxyviolacin have identified three alkyl-oxindoles related to violacin as possible intermediates. Indirubin (29) was first isolated in 1986 from plasma, urine, and hemofiltrate in human blood, and in 2001 from the leaves of *Isatis tinctoria*, its isotopes, isoindirubin (30) and isoindigo (31), were isolated together.^48–59^

## 4. Synthesis of spiro oxindoles & related natural products

### 4.1 Construction of oxindole-based synthetic products

**Scheme 1:**
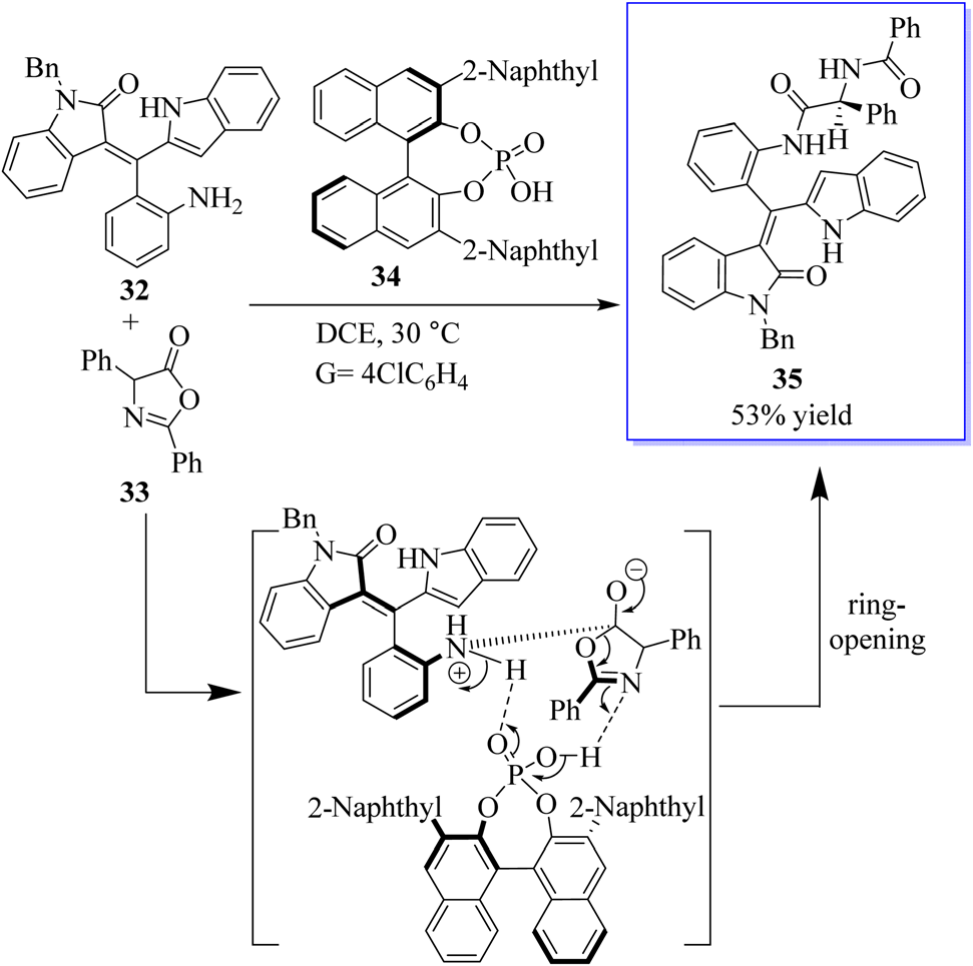
Construction of the tetracyclic core of lycorine-type alkaloids and its application in the formal synthesis of α-lycorane.

The atroposelective synthesis of axial chiral molecules has attracted considerable attention from chemists due to the importance of these molecules. However, due to the low rotational barriers and low configuration stability of these molecules, the catalytic asymmetric synthesis of axial chiral styrene or vinyl arene is underdeveloped and difficult. Therefore, the development of powerful strategies for the selective catalytic synthesis of axial cyclic or vinyl arenes is extremely important. In one study, the first selective access to the axially chiral styrene based on oxindole by a catalytic kinetic resolution strategy was developed. This strategy provides two types of oxindole-based axially chiral derivatives of styrene with good diastereoselectivity (94 : 6dr) and excellent enantioselectivity (98% ee) with high selectivity factors (*S* up 106). This strategy not only facilitates access to the axially chiral styrene based on oxindole, but also provides a robust method for the synthesis of bisamide derivatives 35 with both axial and central chirality. More importantly, this strategy added new members to the axially chiral styrene family (Scheme 1).^60^

**Scheme 2:**
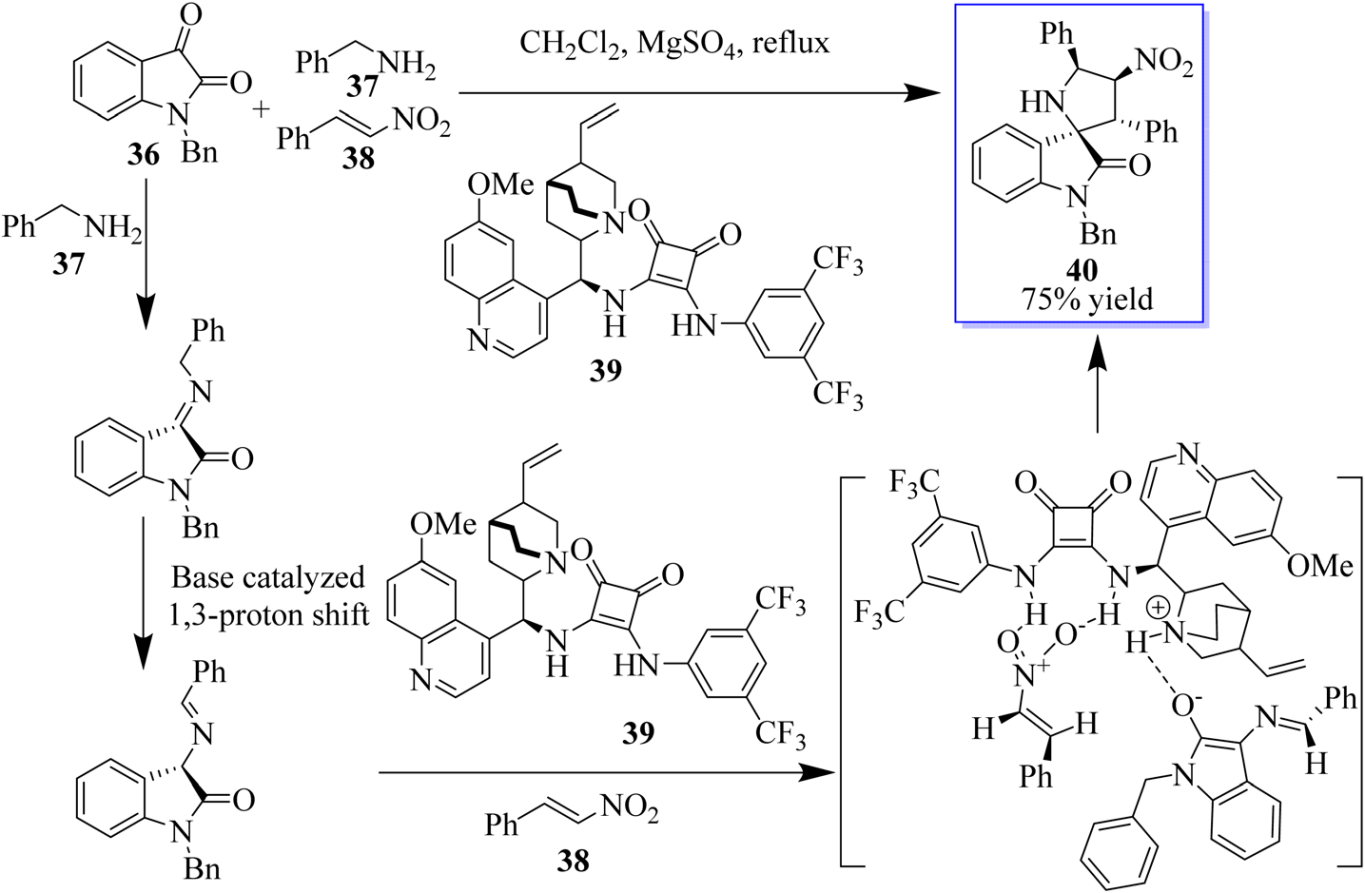
Catalytic enantioselective 1,3-proton shift/[3 + 2] cycloaddition for the synthesis of spirooxindoles.

The 3,3′-pyrrolidinylspirooxindole scaffold is a privileged structural motif that can be found in a wide range of natural products and pharmaceuticals, which possesses various biological activities, such as antitumor, antidiabetic, anti-inflammatory, and antitubercular activities, among others. Because of these significant bioactivities, 3,3′-pyrrolidinylspirooxindole has emerged as an attractive target, and some elegant strategies for its construction have been established. An enantioselective approach to assemble heterocycle 40 involving a three-component reaction of isatins 36, amines 37, and nitroalkenes 38 catalyzed by chiral bifunctional squaramide 39 is well documented in this direction (Scheme 2).^36^

**Scheme 3:**
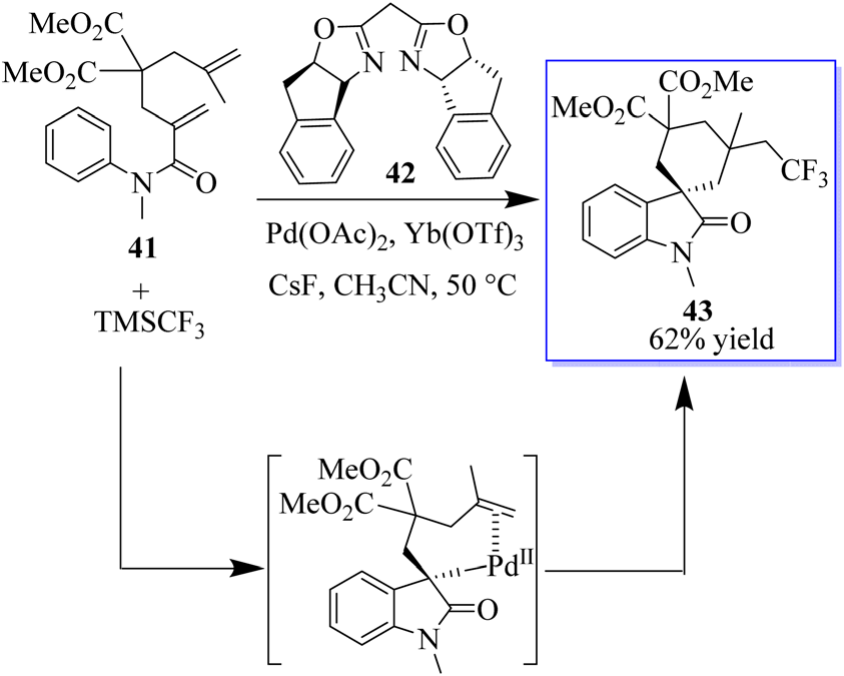
Pd-catalyzed aryl trifluoromethylation of alkenes.

The oxidative aryl trifluoromethylation reaction of activated alkyls catalyzed by palladium was studied in intramolecular and molecular oxidation reactions. The reaction allows the efficient synthesis of various CF_3_-containing oxides 43 in moderate to good yield. The preliminary mechanical studies indicated that the reaction involves an intermediate of Csp^3^Pd^IV^(CF_3_), which is reduced to enable he formation of Csp^3^CF_3_ bonds (Scheme 3).^61^

### 4.2 Construction of the oxindole-based natural products

**Scheme 4:**
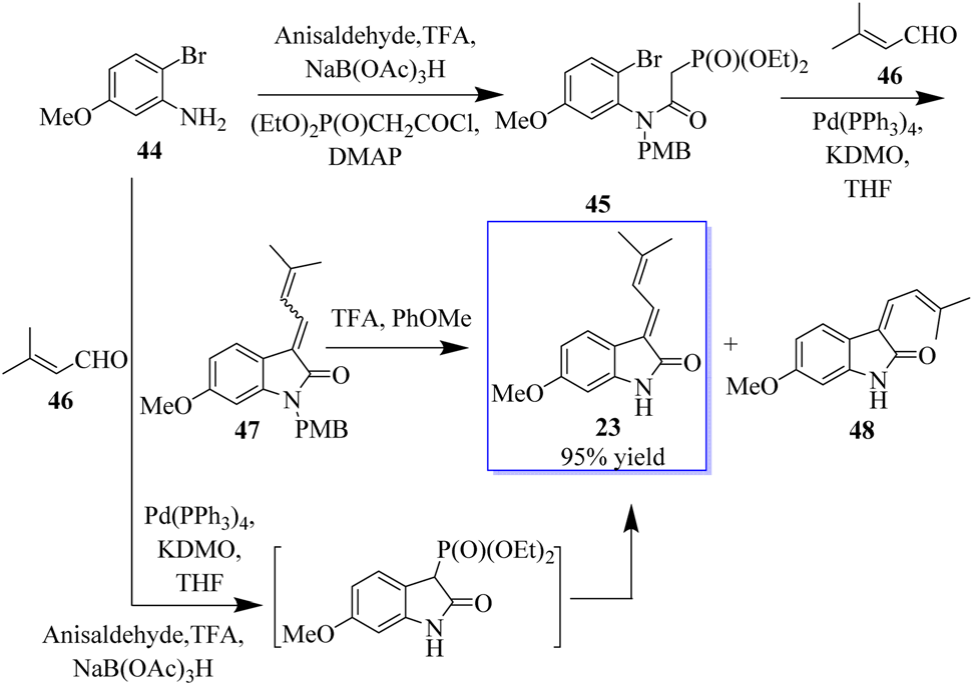
Synthesis of *E* and *Z*-soulieotine.

The enolate arylation/HWE sequence was also used for the first synthesis of the simple natural product soulieotine (23) (Scheme 4). Soulieotine was isolated from the rhizomes of *Souliea vaginata*, which is used as an anti-inflammatory and analgesic plant in traditional Chinese medicine. The PMB-protected cyclization precursor was easily obtained from aniline and using 3′-methylbutene as a trapping agent gave the expected conductor, with an unoptimized yield as an isomer mixture of alkene. It should be noted that enolate arylation using Pd(OAc)_2_ was not successful, probably because of the deprotection of PMB mediated by palladium(ii) but proceeded with tetrakis(triphenylphosphine)palladium. After TFA protection, the mixture of *E* and *Z*-soulieotine (2 : 1) was separated by chromatography.^62^

**Scheme 5:**
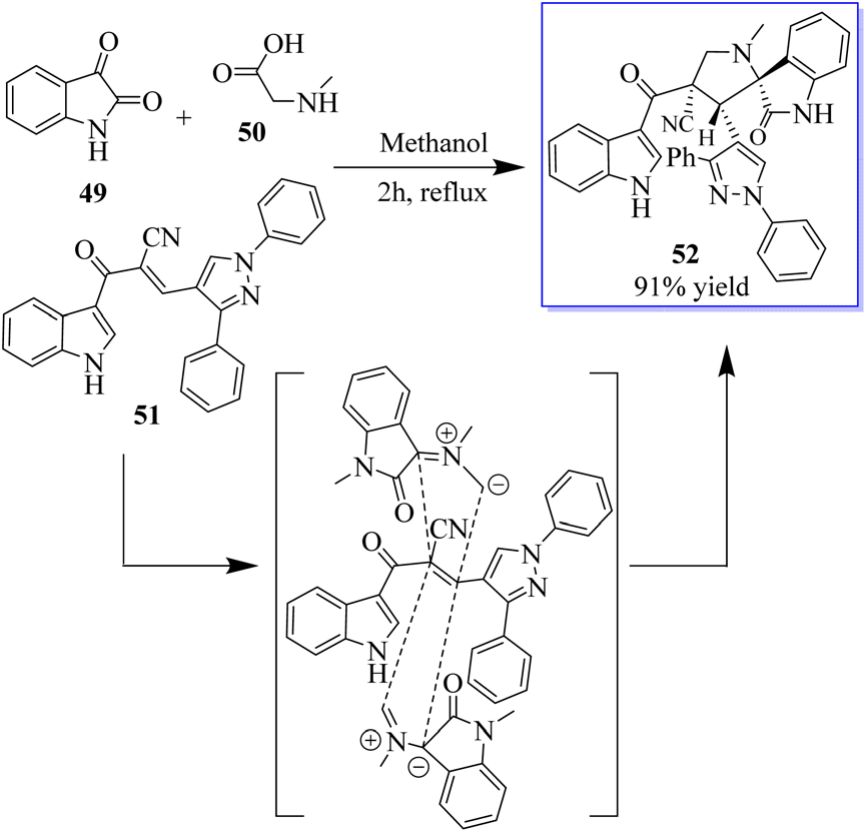
Synthesis of spiropyrrolidine-oxindoles by [3 + 2] cycloaddition.

**Scheme 6:**
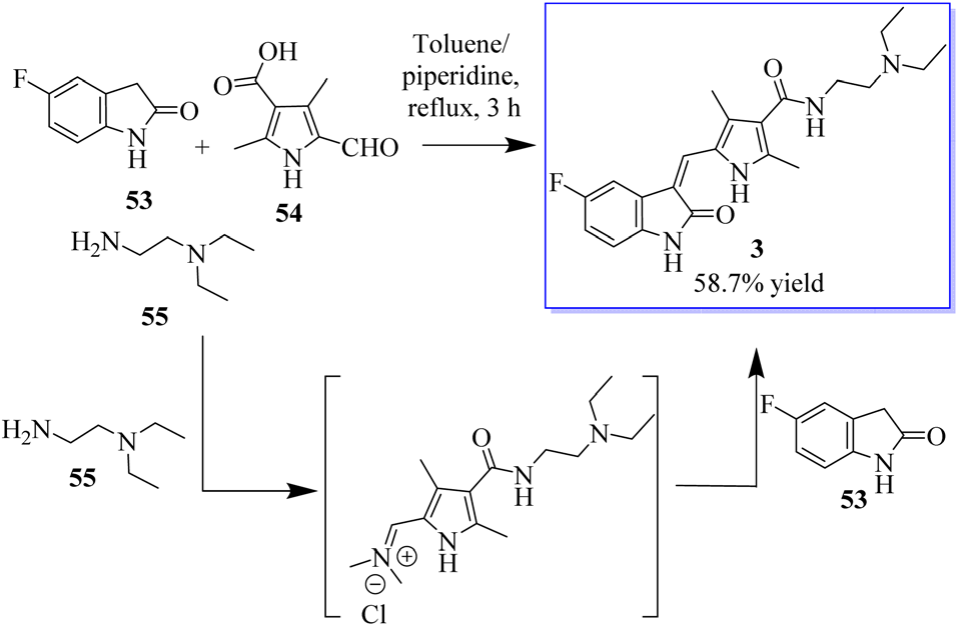
Synthesis of sunitinib.

Under the optimum conditions, a series of novel highly functionalized spiropyrrolidine-oxindoles 52 was synthesized *via* the 1,3-dipolar-cycloaddition of azomethine ylides derived from isatine 49 and various amino acids such as sarcosine, phosphate, and typroline, and dipolarophile (1,3-diphenyl-1*H*-pyrazol-4-yl)-2-(1*H*-indole-3-carbonil)acrylonitriles. All synthetic compounds have been evaluated for antimicrobial activity and showed significant activity (Scheme 5).^63^ The process involves converting 5-fluoroindolin-2-one, 5-formyl-2,4-dimethyl-1*H*-pyrrole-3-carboxylic acid and its derivatives, and *N*^1^,*N*^1^-diethylethane-1,2-diamine to give sunitinib 3. Sunitinib produced by the above-mentioned process has only 93.87% purity, as shown in Scheme 6.^64^

**Fig. 6:**
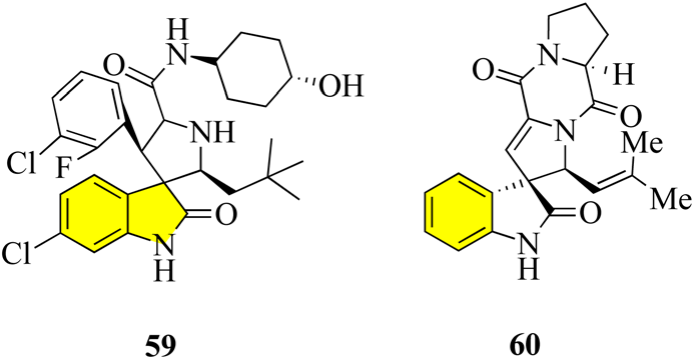
Bioactive molecules containing spiro-[pyrrolidin-2,3′-oxindole].

**Scheme 7:**
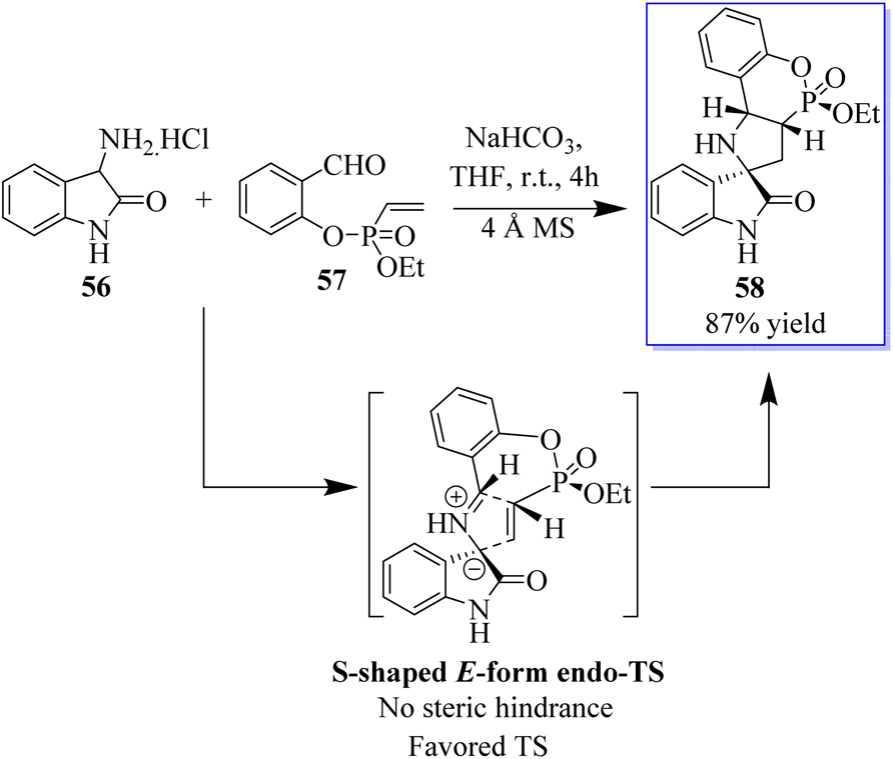
Synthesis of oxindole spiro-*P*,*N*-polycyclic heterocycles.

Pyrrolidin-2,3′-oxindoles 59–60 are representative spirooxindole compounds, which are notable heterocyclic frameworks because they are widespread in many natural products and synthetic compounds. They have a wide range of bioactivities such as anticancer, antibacterial, and MDM_2_ inhibitory effects (Fig. 6).^65^ A reliable stereoselective assembly strategy for the construction of pyrrolidin-2,3′-oxindole *cis*-fused phosphadihydrocoumarin 58 was established. This process involves the condensation of *O*-vinyl-phosphate salicylic aldehydes and 3′-aminooxindoles, followed by intermolecular cycloaddition with high diastereoselective and atom economy (Scheme 7).^66^

## 5. Chemical reactivity of 2-oxindole and related derivatives in the presence of chiral catalysts

**Scheme 8:**
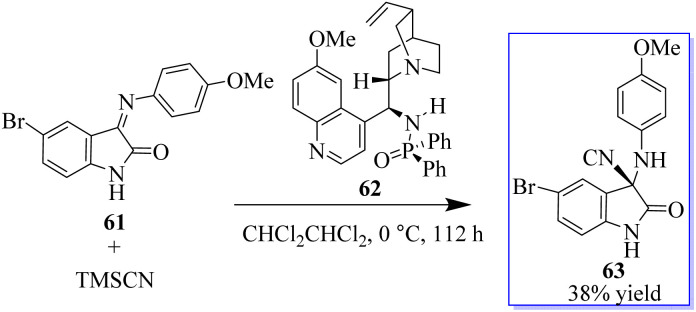
Isatin ketimine asymmetric Strecker reaction.

Quinine-based catalysts 62 were first applied in the Strecker reaction of *N*-aryl isatin ketimines 61 with trimethylsilyl cyanide (TMSCN) (Scheme 8) because at that time, the bifunctional tertiary-amine catalyzed Strecker reaction was undeveloped,^67^ although Deng had reported a highly enantioselective chiral tertiary-amine mediated ketone cyanosilylation to produce 63.^68^ Preliminary studies indicated that 10 mol% phosphinamide catalyst afforded oxindole based on an aminonitrile.^69,70^ Despite the unsatisfactory result, the phosphinamide catalyst afforded clearly better enantioselectivity than analogous catalysts with amide or thiourea as the H-bond donor.

**Scheme 9:**
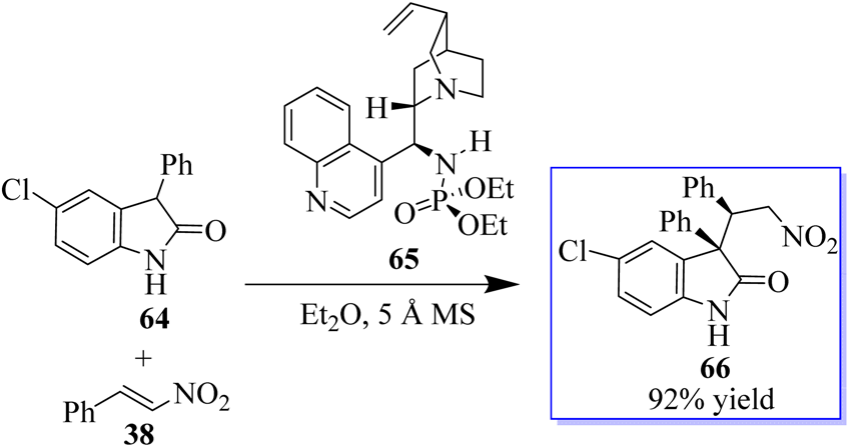
Asymmetric Michael addition.

**Scheme 10:**
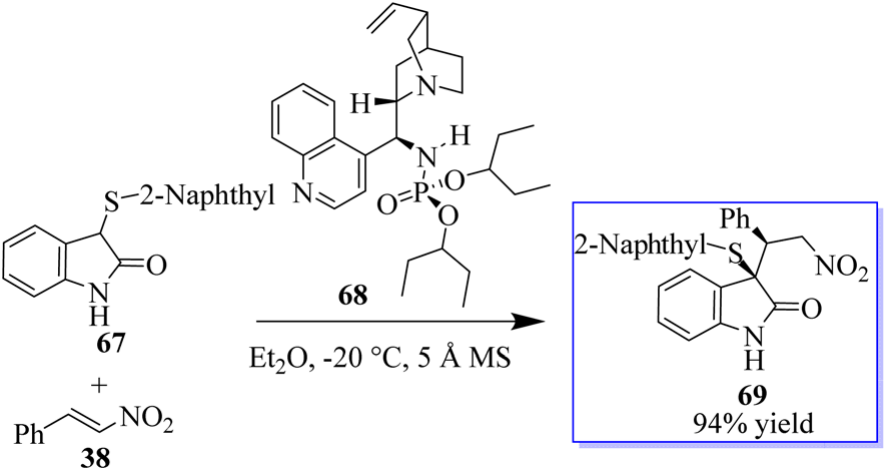
3-Thioxindole addition by Michael reaction.

This result exhibited their potency in other reactions and triggered subsequent research on the catalytic enantioselective addition of nucleophiles to isatin ketimines for the synthesis of chiral 3-substituted aminooxindoles. The bifunctional phosphoramides were subsequently found to be potent catalysts for the Michael addition of 3-substituted oxindoles 64 to nitroolefins 38 (Scheme 9).^71–73^ The resulting adducts are valuable synthons to access quaternary oxindole 66 and indoline derivatives; however, previous studies relied on the use of highly active *N*-protected 3-substituted oxindoles and had limited substrate scope. Unprotected 3-substituted oxindoles were less reactive but more convenient and atom-efficient to prepare. The simple and easily available cinchonidine-derived phosphoramide is used to achieve high to excellent diastereo- and enantioselectivity. Notably, both 3-aryl- and 3-alkyloxindoles as well as aryl- and alkyl-substituted nitroolefins are viable substrates, giving the desired quaternary oxindoles with excellent enantioselectivity. Later, with phosphoramide having a bulky ester group, the highly enantioselective Michael addition of 3-alkylthio- and 3-arylthiooxindoles was developed, giving various 3-substituted 3-thiooxindoles 69 in high yield (Scheme 10). This reaction could be run on a gram scale with only 1.0 mol% catalyst 68.^74*a*^

**Scheme 11:**
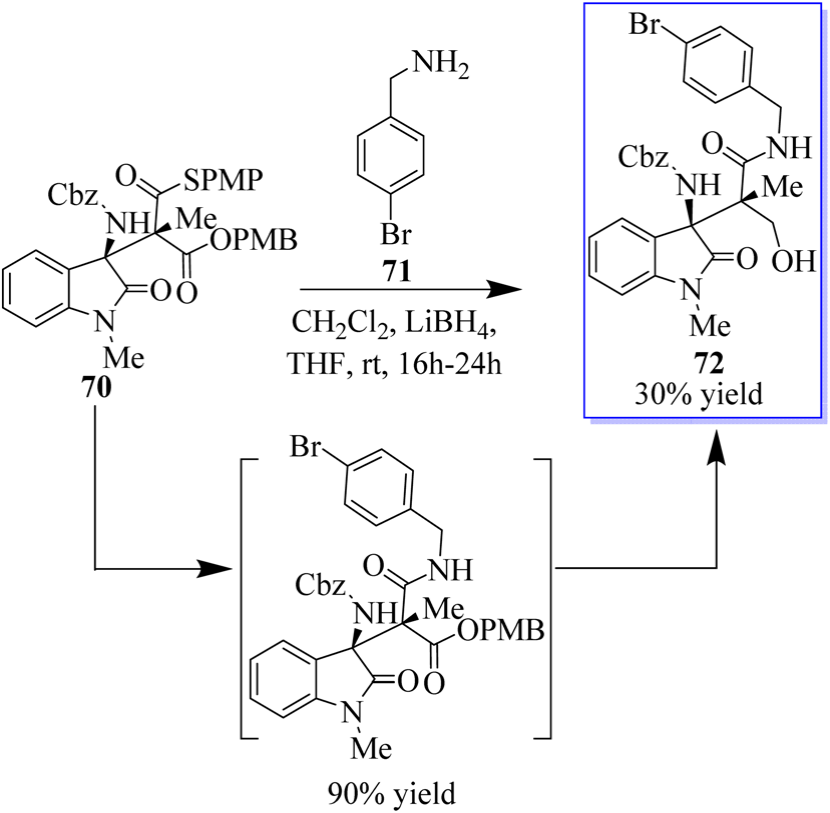
Functionalization of the thioester and oxo-ester moieties.

The oxides with adjacent tetrasubstituted stereocenters were obtained in moderate yields and stereoselectivities by the monothiomalonate (MTM) monocatalyzed conjugate addition reaction of *N*-Cbz ketimines derived from isatin. This method requires 2 mol% catalyst load and operates under moderate reaction conditions. Both enantiomers can be used with Takemoto's catalyst or an alkaloid derivative of cinchona, with good yields and excellent stereoselectivities. The synthetic methodology allowed a direct route to the derivatives of the antagonist of the agonist of the gene/cholecystokinin-B receptor AG-041R. The reactivity of the connected functional groups can be decreased by molecular crowding at nearby carbon atoms that are fully substituted. In this reaction, the authors treated ent-70 with 4-bromobenzylamine 71, which solely interacted with the thioester group to afford the amide (intermediate), to test if the differentially accessible oxo and thioester moieties could be further functionalized. Alcohol 72 was produced by further selective reduction of the oxoester moiety, which crystallised, allowing the unambiguous assignment of the absolute and relative stereochemistry of the addition products (Scheme 11).^74*b*^

**Scheme 12:**
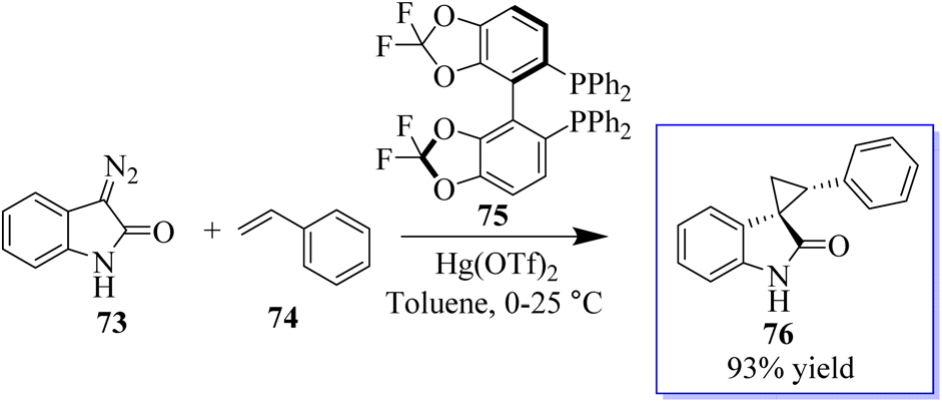
Asymmetric olefin cyclopropanation reaction catalyzed by Hg(ii).

The first known instance of diazo compound-mediated Hg(ii)-catalysed olefin cyclopropanation was revealed. Spirocyclopropyl oxindoles can act as donor–acceptor cyclopropanes for complexity-generating synthesis,^75^ in addition to being useful pharmacophores. In this reaction, enantioselective cyclopropanation to obtain 76 was revealed using chiral difluorophos 75 in conjunction with Hg(OTf)_2_ (Scheme 12).^76^

**Scheme 13:**
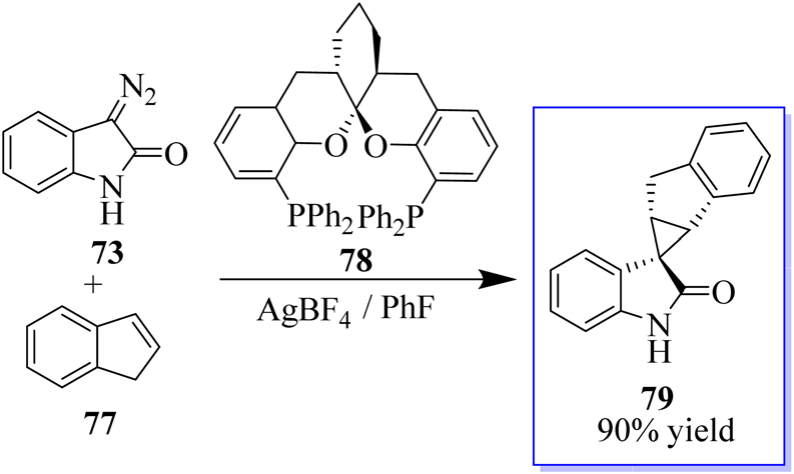
Cyclopropanation of olefins catalyzed by Au(i).

To produce cyclopropanes with high enantioselectivity, both unprotected and *N*-methyl diazooxindoles performed well. Ligand acceleration effects were observed because using 0.4 equivalent of chiral ligand in comparison to Hg(OTf)_2_ produced comparable results. Furthermore, changing the counter anion improved the catalytic properties, where difluorophos/Hg(OTf)_2_ failed in these reactions, whereas difluorophos/Hg(PF_6_)_2_ achieved high activity, albeit with moderate enantioselectivity, in the cyclopropanation of disubstituted olefins. These findings demonstrated that ligands could be used to modify the catalytic properties of mercury dioxide (Hg(ii)). The cyclopropanation of di- and trisubstituted olefins was then performed with high enantioselectivity using an Au(i) catalyst. In fact, it is challenging to develop a general catalyst for the full control of stereoselectivity in the cyclopropanation of *trans*- or *cis*-1,2-disubstituted and trisubstituted alkenes due to the high sensitivity of metallocarbenes to the steric hindrance and geometry of alkenes. Potent catalyst ((S,S,S)-SKP L1) 78 enabled highly stereoselective cyclopropanation with a variety of alkenes, including monosubstituted, *cis*- and *trans*-1,2-disubstituted, 1,1-disubstituted, and even trisubstituted alkenes, which is Ding's spiroketal bisphosphine-derived complex (Scheme 13).^77^

**Scheme 14:**
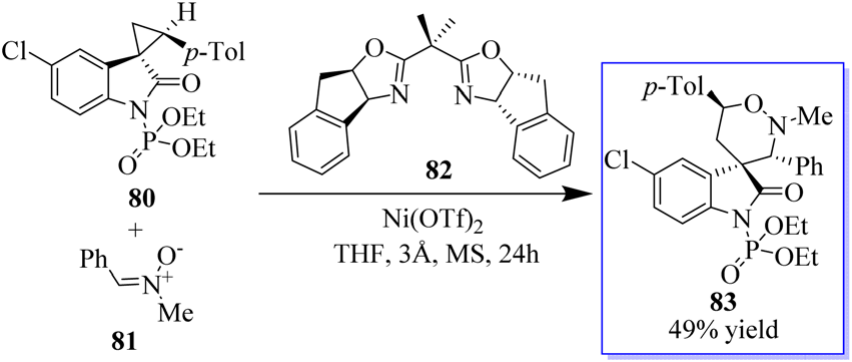
[3 + 3] cycloaddition of nitrones in an enantioselective manner.

Adjusting the electron-withdrawing *N*-protecting group, the synthesis of spirocyclopropyl oxindoles was revealed. The activation by an appropriate Lewis acid produced *N*-diethoxyphosphoryl oxide 80, which can be converted into spirocyclic oxindoles 83 and 3,5-disubstituted pyrrolidinone by [3 + 3] cycloaddition with nitrone 81, cyclization with 1,4-dithiane-2,5-diol, and ring opening/cyclization with primary amine. Under Cu(OTf)_2_ catalysis, the *N*-benzoyl oxindole performed better in the [3 + 2] cycloaddition with aldehyde (Scheme 14). The absence of these transformations in the presence of unprotected or *N*-methyl spirocyclopropyl oxindoles proves the unmistakable activation effects of *N*-protecting groups. This activation strategy works well to produce oxindole-based spirocyclic tetrahydro-1,2-oxazine in catalytic enantioselective reactions using spirocyclopropyl oxindoles. Surprisingly, acetophenone-derived ketonitrones are also good substrates and can be used to make spirooxindoles with nearby quaternary and tetrasubstituted carbon stereocenters. Notably, this is the first method to use the inactivated ketonitrones in enantioselective catalytic synthesis; however, this field has not received much attention.^78–83^

**Scheme 15:**
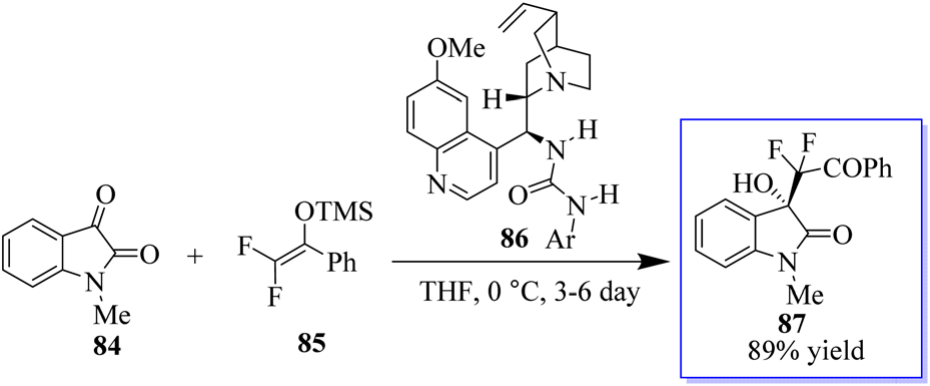
Reaction of difluoroenoxysilanes *via* Mukaiyama-aldol reaction.

It is common practice to modify the pharmaceutical properties of organic molecules by selectively adding a fluoroalkyl group. Consequently, oxindoles with a fluoroalkyl group at the C-3 position make intriguing targets for the creation of pharmaceuticals and biological probes. However, although enantioselective trifluoromethylation has been extensively investigated, enantioselective mono- or difluoroalkylation techniques have not.^84,85^ The creation of chiral carbons with an easily accessible mono- or difluoromethylated ketone moiety *via* selective fluoroalkylation^86^ is challenging. It has been revealed that amines can activate fluorinated silyl enol ethers (FSEEs) for enantioselective synthesis with the bifunctional tertiary-amine-catalyzed Strecker reaction using TMSCN. Consequently, the highly enantioselective Mukaiyama-aldol reaction involving isatin 84 and difluoroenoxysilane 85 was catalyzed by urea derived from quinine 86, producing 3-hydroxyoxindoles 87 (Scheme 15).^87^

**Scheme 16:**
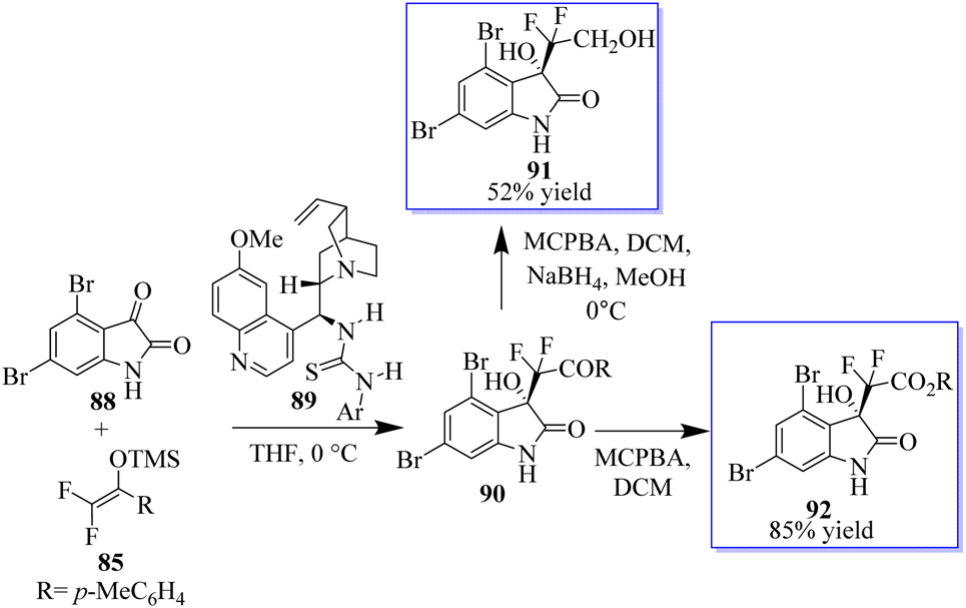
Synthesis of convolutamydine difluoro analogue.

Given that the configuration of convolutamydines is *R*, they are generated by the reaction of isatin 88 with olefin 85 in the presence of quinidine-based catalyst 89. The difluoro analogue of convolutamydine E (91) was easily produced in 52% yield after the Baeyer–Villiger oxidation of 90 generated 92 in 85% yield without the loss of ee (Scheme 16).^19,88–90^

**Scheme 17:**
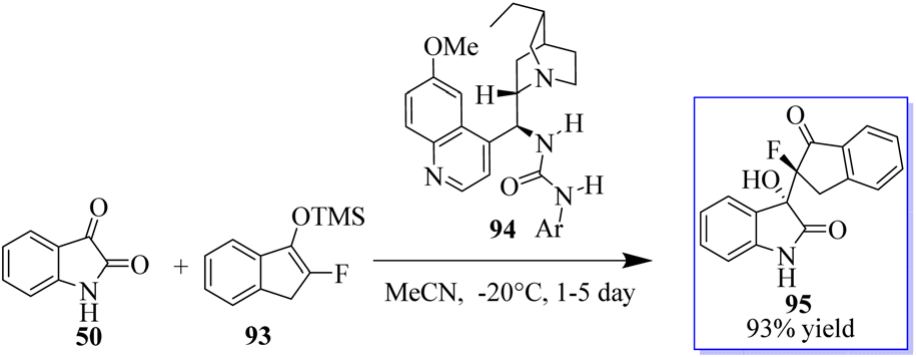
Mukaiyama aldol reaction of isatins.

According to Liu, the reaction of isatins 50 and 93 catalyzed by 94 generates a quaternary chiral centre at the C-3 position of oxindole. Even after 3 days, the reaction in THF at 20 °C moved slowly, producing the desired product 95 in 95% yield and with modest stereoselectivities (Scheme 17).^91^

**Scheme 18:**
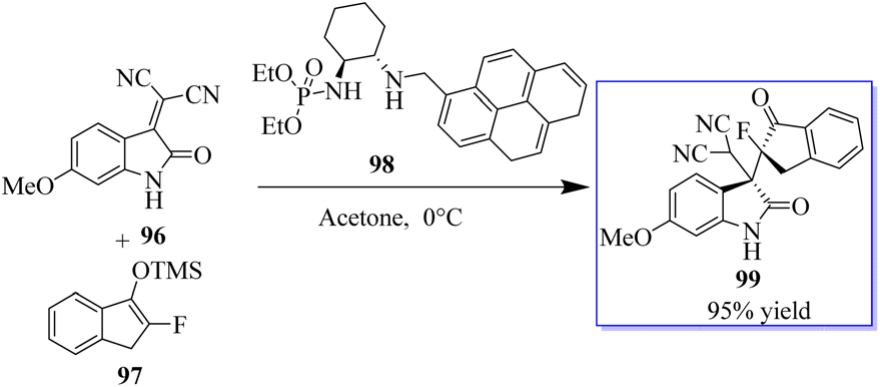
Mukaiyama–Michael addition reaction.

**Scheme 19:**
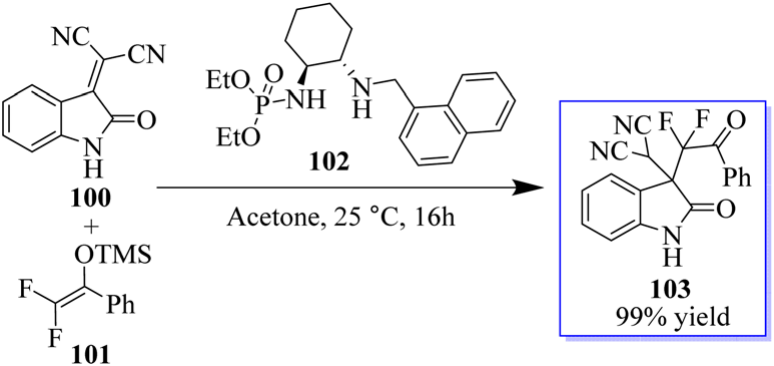
Implications of phosphoramide.

Monofluorinated enol silyl ethers 97 derived from either an α-fluoroindanone or benzofuranone were also effectively activated by the chiral secondary amine phosphoramide 98 for reaction with isatylidene malononitrile 96 to produce adjacent and fully substituted carbon stereocenters. However, in this instance, 98, which had a 1-pyrenylmethyl group on the secondary amine moiety, had the best outcome. Monofluorinated oxindole derivatives 99 were produced under these reaction conditions in excellent yield with high to excellent dr and ee values (Scheme 18).^92–94^ Surprisingly, catalyst 102 enabled the reaction between 100 and 101 to be completed in about 16 h at room temperature with acetone as the solvent, producing 103 in 99% yield (Scheme 19).^94^

**Scheme 20:**
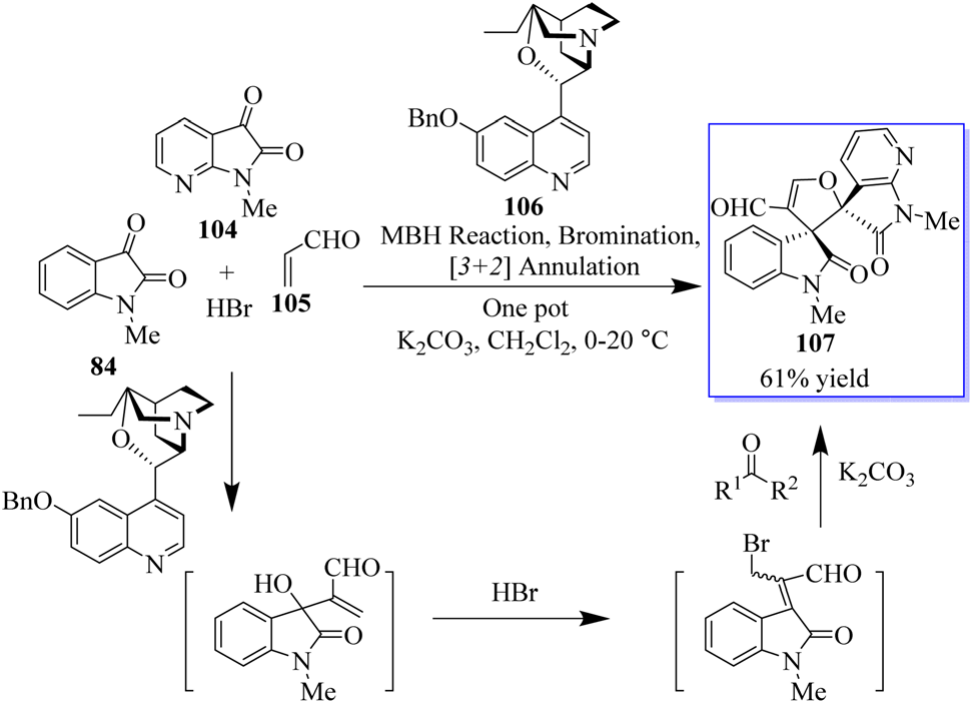
Triple asymmetric sequence.

**Scheme 21:**
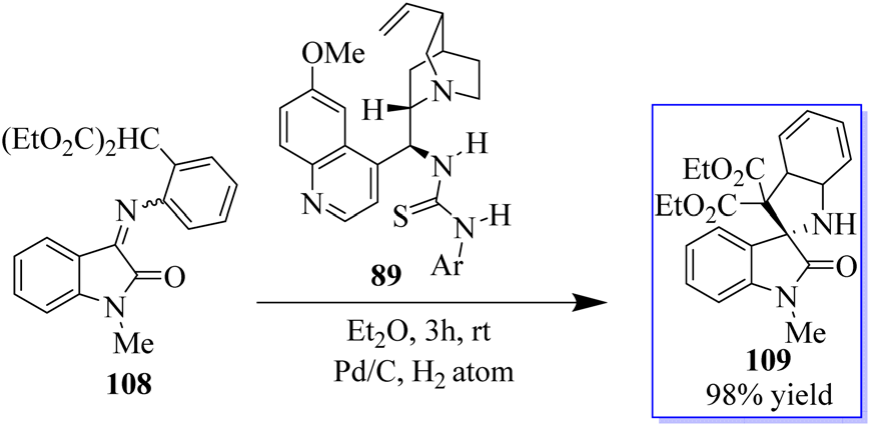
Catalysis of asymmetric triple sequence.

A novel MBH/bromination/annulation sequence made up of three intermolecular reactions to build on the highly enantioselective Morita–Baylis–Hillman (MBH) reaction of isatins 84 and acrolein 105 has been developed. The oxindole-based tetrasubstituted alkenes were produced by the tertiary-amine-catalyzed MBH reaction, and then they underwent a highly stereoselective [3 + 2] annulation with a variety of activated ketones to produce bis(spiro)oxindoles 107 and spirocyclic oxindoles with adjacent quaternary/tetrasubstituted carbon (Scheme 20).^95–97^ Using 10 mol% of bifunctional thiourea 89 produced from quinine in Et_2_O at room temperature, the range of the asymmetric 6π electrocyclization was investigated. The required spirocyclic oxindole derivatives 109 were produced in good yield by a variety of malonate–ketimines 108 with various substituents on the isatin framework (Scheme 21). This process effectively combined the hydrogenation of nitrobenzene by Pd, keta-imine formation by Brønsted acid, and asymmetric electrocyclization by bifunctional tertiary amine in one step. Because the one-pot process avoided racemic cyclization of malonate–anilines during the purification by column chromatography and significant yield losses linked to the purification of malonate–ketimines 108, the synthetic efficiency significantly increased. Also, the background electrocyclization was suppressed and the bifunctional tertiary-amine-mediated enantioselective reaction was not adversely affected by using only 4 mol% TsOH to promote ketimine formation.^98,99^

**Scheme 22:**
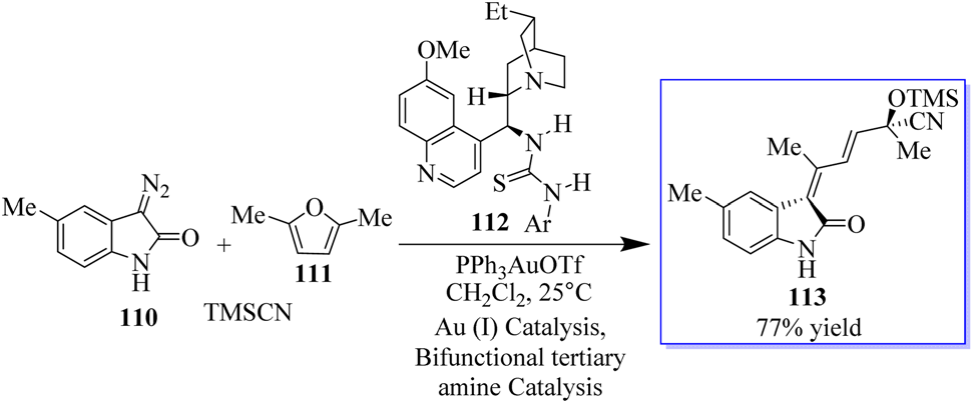
Tertiary amine nucleophilic catalysis with Au(i).

Chiral tertiary amine 112 serves as a Brønsted base to deprotonate and activate the nucleophilic reaction partner of the Michael addition or amination reaction, while the Au-catalyzed reaction provides the 3-substituted oxindole as the nucleophile. As an alternative, the synthesis of chiral 3-alkenyloxindoles 113 from diazo-oxindoles 110, disubstituted furans 111, and TMSCN was highly enantioselective due to the integration of Au-catalyzed enone formation and tertiary-amine-mediated cyanosilylation of ketones (Scheme 22).^100^

**Scheme 23:**
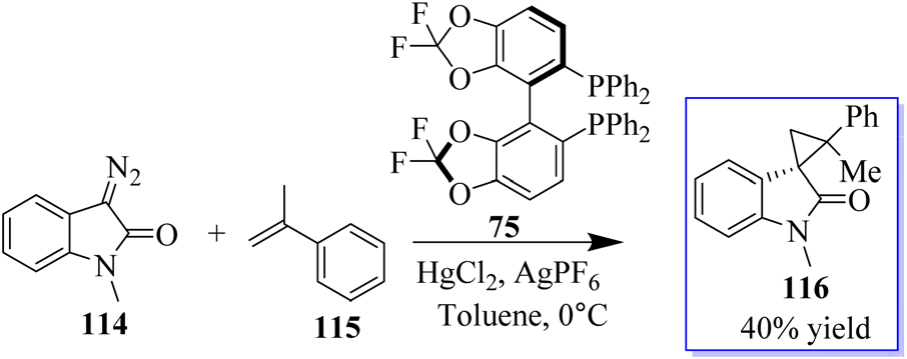
Cyclopropanation of diazooxindole and styrene.

Diazooxindoles are multifunctional cyclic diazo reagents for the diversity-oriented synthesis (DOS) of substituted oxindoles through reagent-controlled catalytic diversification, including insertion, cyclopropanation, and cycloaddition reactions. Diazooxindoles 114 are easily produced from isatins on a large scale. When the counteranion was changed from OTf to PF6, the less reactive disubstituted alkenes were also suitable substrates for this reaction. Consequently, when 5.0 mol% of *in situ*-created catalyst 75/Hg(PF6)_2_ was utilised, *R*-methylstyrene 115 produced 116 in moderate yield, with two neighbouring quaternary stereogenic carbon centres (Scheme 23).^76^

## 6. Miscellaneous reactivities of 2-oxindole and related derivatives

**Scheme 24:**
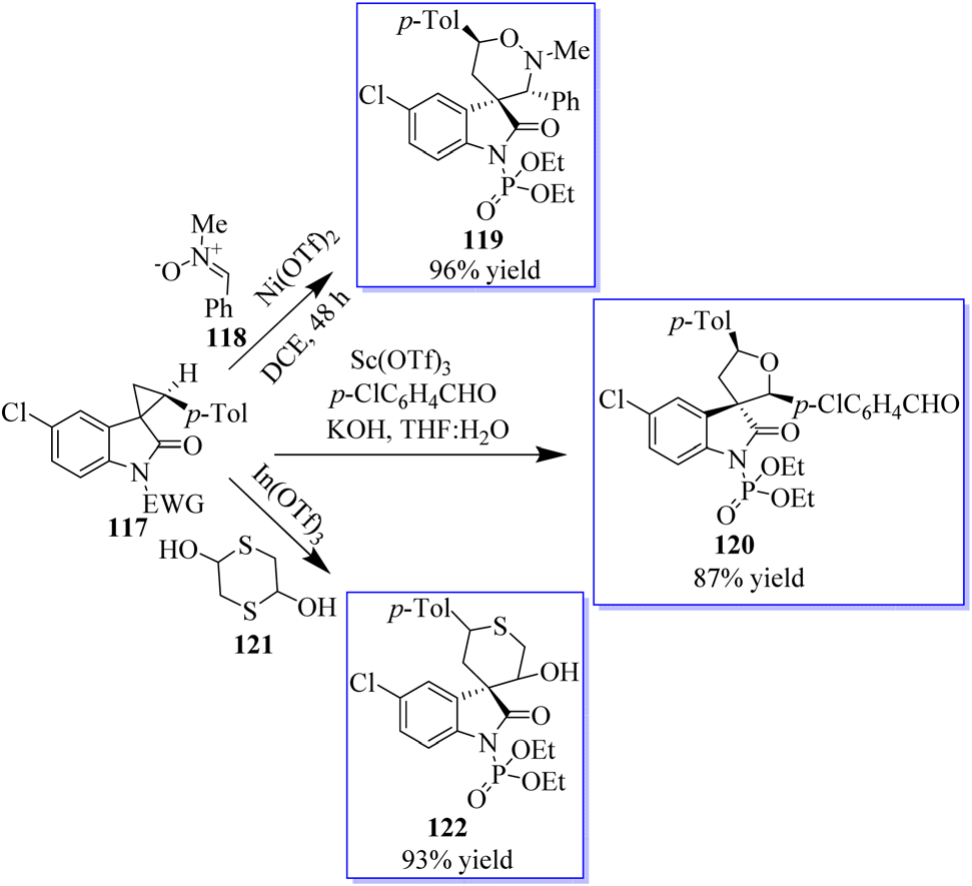
Spirocyclopropyl oxindoles-based DOS (diversity-oriented synthesis).

Although protocols were restricted to substituted cyclopropane-1,1-dicarboxylates or diketones, the cycloaddition of doubly activated cyclopropanes is a successful method for the enantioselective synthesis of cyclic compounds. A particular variety of mono-activated cyclopropane is spirocyclopropyl oxindoles. The low reactivity of these substances prevents the development of new reactions and the associated catalytic enantioselective studies, whereas Carreira invented and demonstrated the value of their cycloaddition with imines. A method for activating spirocyclopropyl oxindoles 117 using an *N*-protecting group that draws electrons was described. This modification allows for the bidentate coordination of oxindoles to chiral metal complexes for improved enantiofacial control and effectively stabilizes the negative charge developed at C-3 of an oxindole through charge separation upon the activation of a Lewis acid (Scheme 24).^78,81,82^ Due to the superiority of cationic Au(i) catalysis in the olefin cyclopropanation of diazooxindoles, the advantage is the previously unrecognized sequential Au(i)/chiral tertiary amine catalysis as an alluring method to develop diversity-oriented asymmetric tandem reactions, enabling the quick creation of scaffold diversity from diazooxindoles. The success of these tandem reactions, brought about by the high activity of cationic Au(i) catalysis, allowed the use of only 1.0 mol% gold complex to realize these transformations of diazooxindoles, although cationic Au(i) catalysis is known to be incompatible with tertiary amine catalysts. The performance of chiral tertiary amines, which are used at a 10 mol% concentration, is not significantly impacted by the remaining gold catalyst.

**Scheme 25:**
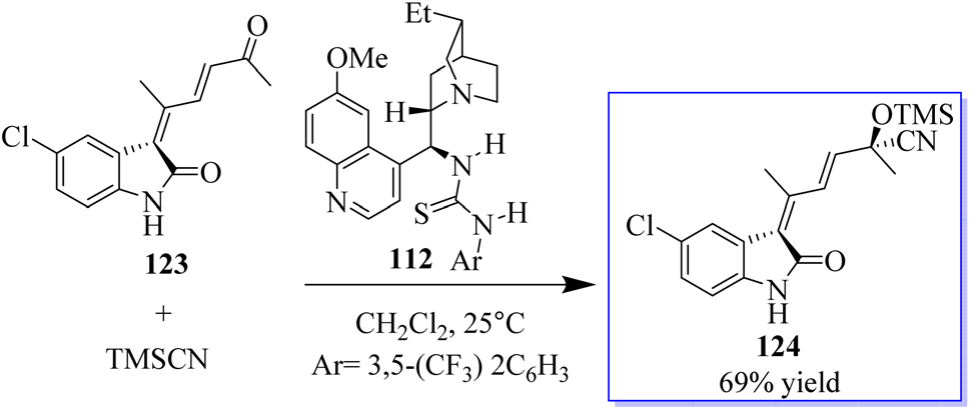
Catalysis of tertiary amine by asymmetric Au(i).

The successful synthesis of multifunctional enone 123 paved the door for the one-pot synthesis of chiral 3-alkenyloxindoles by combining gold-catalysed enone production with catalytic enantioselective addition of a nucleophile to the carbonyl group of 123. Based on our findings in the Strecker reaction employing TMSCN catalysed by bifunctional tertiary amines, the authors initially attempted the asymmetric cyanosilylation of enone. Fortunately, bifunctional quinine-derived squaramide or (thio)urea 112 could mediate this reaction, and up to 69% of the desired product 124 was produced when 112 was utilised but the reaction progressed slowly even with a 20% catalyst at 25 °C (Scheme 25).^100^

## 7. Miscellaneous synthetic approaches for 2-oxindole and related heterocycles

**Scheme 26:**
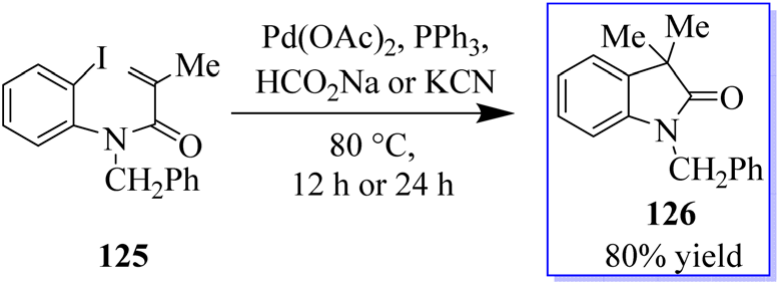
Pd-catalyzed carboiodination reaction.

The palladium-catalyzed Mizoroki–Heck reaction has been a staple in the arsenal of organic chemists ever since it was discovered in the 1970s. A C–X electrophile is added oxidatively in this reaction, and followed by 1,2-migratory insertion. Base-mediated HX reductive elimination regenerates the Pd(0) catalyst, while a subsequent β-H elimination produces the unsaturated cross-coupled product. The suppression of β-H elimination has historically been accomplished in several ways, including electronic and steric bias of the catalyst or the production of a neopentyl organometallic species devoid of β-hydrogens. By using a tethered disubstituted olefin in an intramolecular 6-*endo*-trigtype cyclization of 125, the Grigg group developed a method to disrupt the typical Mizoroki–Heck-type mechanism in the early 1990s (Scheme 26).^101–104^

**Scheme 27:**
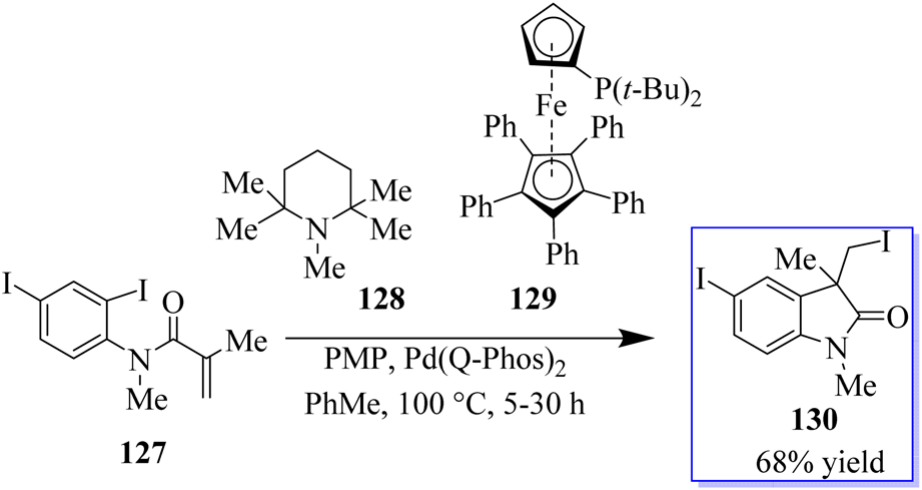
Polyiodinated compounds employed in the carboiodination reaction.

In a migratory insertion, a neopentyl species is formed, which is incapable of undergoing β-H elimination in the presence of Pd(Q-Phos)_2_129 and 1,2,2,6,6-pentamethylpiperidine 128 in toluene at 100 °C. Under these conditions, 130 was isolated in 68% yield. With the optimized reaction conditions in hand, the authors examined a series of diiodinated compounds 127. By extending these findings to a one-pot multistep reaction where both the carboiodination reaction and a conventional palladium-catalyzed Mizoroki–Heck reaction on the other halogen moiety were carried out, it showed that these scaffolds could be used as an “oxindole linchpin” molecule in organic synthesis (Scheme 27). Product 130 from both the intramolecular carboiodination reaction and the intermolecular Mizoroki–Heck reaction were identified by *in situ* NMR analysis of these reactions (precyclization).^100^ According to these findings, the catalyst may be able to reversibly add to each carbon–iodine bond, enabling the two reactions to take place simultaneously with perfect selectivity.^105^

**Scheme 28:**
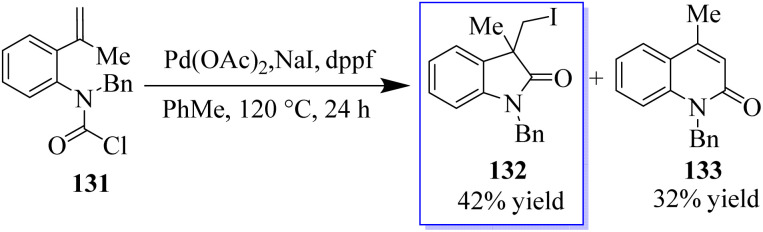
Enantioselective carbamoyl iodination reaction catalyzed by nickel.

Fortunately, five members of benzo-fused lactam 132 were also easily isolated in 42% yield when compound 131 was used as a substrate in these reaction conditions. In addition to the carboiodination product 132, Heck reaction product 133 was obtained with a yield of 32% probably by the 6-end cyclization method (Scheme 28).^106^

**Scheme 29:**
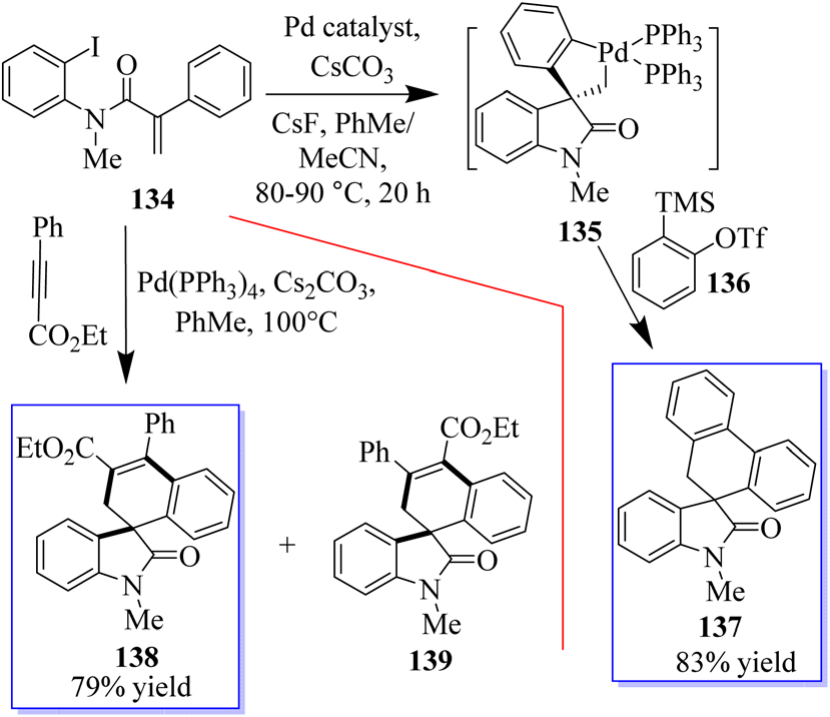
Synthesis of Pd-catalyzed spirocyclic oxindole.

**Scheme 30:**
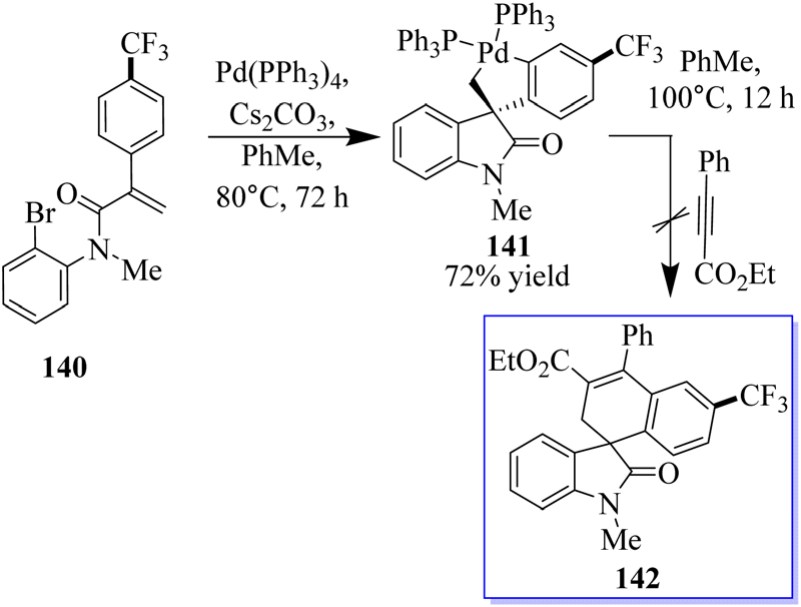
Synthesis of Pd-catalyzed spirocyclic oxindole: potential mechanisms.

When substrate 134 reacts with the precursor of benzene 136 in MeCN 90 °C with CsF and a Pd-(OAc)_2_/PPh_3_ (OAc) catalyst, a small proportion of the expected product 137 was formed. Subjecting 134 to these reaction conditions gave trace amounts of 138 and 139. The spirocyclic structure 138 was confirmed by spectroscopy and X-ray crystallography of single crystals. Taking advantage of this initial result, the reaction conditions were optimized to produce 138 in a yield of 79% and minimize the formation of side products (Scheme 29).^107–109^ With the help of an *o*-haloacrylamide substrate, it was possible to successfully isolate and characterize a spirocyclic oxindole palladacycle. In-depth research was done by the Garcia-Lopez group to clarify how these reactions work (Scheme 30). Palladacycle was synthesized from a halo acrylamide substrate and examined using single-crystal X-ray crystallography. The important five-membered palladacycle was formed after the σ-alkylpalladium complex was treated under C–H metalation conditions. Interestingly, spirocyclic oxindole 142 was produced after the alkylpalladium complex was treated with *in situ*-produced benzyne. This provides evidence for both mechanisms. Inconclusive attempts were made to isolate any intermediates produced when benzyne was inserted into five-membered palladacycle 141. Only the starting material and the final product were visible during NMR (nuclear magnetic resonance) reaction monitoring, indicating that the organometallic intermediates produced by benzyne insertion quickly broke down.^108,109^

**Scheme 31:**
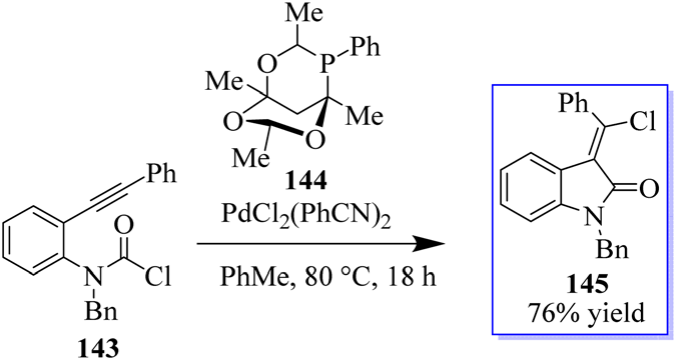
Chlorocarbamoylation reaction of Pd(ii–iv).

Using a PdCl_2_L_2_ catalyst, the chlorination of an alkyne can be achieved *via* chloropalladation reaction. The concurrent cyclization of carbamoyl chloride 143 enabled the highly *Z*-selective production of oxindoles 145 with the help of catalyst PA-Ph 144 (Scheme 31).^110^ Subsequently, the oxindole products were produced by cyclization with carbamoyl chloride through a possible palladium(iv) intermediate. The teams of Hoveyda, Ito, and Yun made significant contributions to this field. Using styrenes containing tethered electrophiles, it is planned to apply this technique intramolecularly to quickly access heterocyclic scaffolds. In this procedure, the authors reported the use of carbamoyl chlorides in a copper-catalyzed cyclization method to synthesize enantioenriched substituted borylated oxindoles 148, continuing our interest in carbamoyl chlorides 146 as electrophiles in metal-catalyzed reactions.

**Scheme 32:**
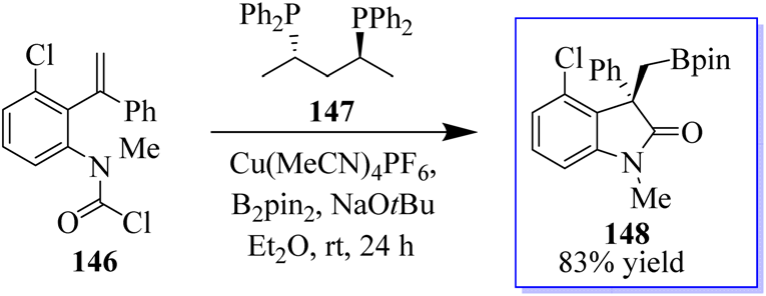
Borylated oxindole production catalyzed by copper.

The active bis(diphenylphosphane) 147 catalyst is used in the enantioenriched oxindole synthesis *via* cyclization into the tethered carbamoyl chloride (Scheme 32).^111^ The chemistry made possible by iodo-oxindoles is complemented by the divergent approach of this methodology to oxindole functionalization. Although conventional boronate oxidation produced the oxidized product, Suzuki coupling of the boronate handle produced the arylated oxindole with no erosion of enantioselectivity. It should be noted that using a strong base caused the hydroxyoxindole product to completely lose its stereochemistry. This is probably because a retro-aldol-oxidation sequence took place. The desired oxidation was achieved with no loss of enantioselectivity by using a milder base.

**Scheme 33:**
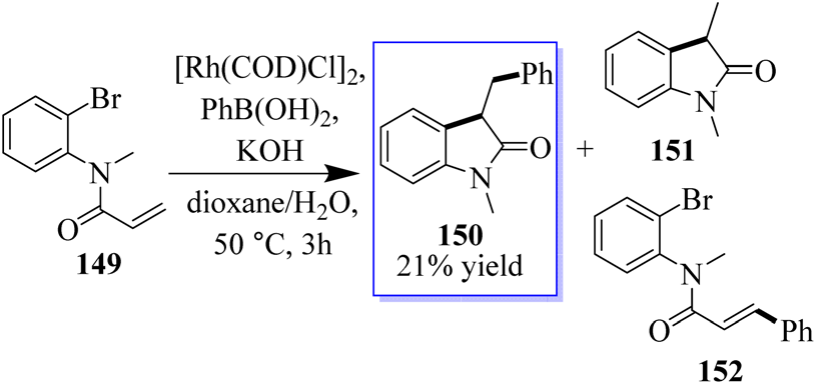
Enolate arylation and domino addition catalyzed by Rh.

Researchers conducted a study on different α-arylation techniques, and in the 1990s, Hayashi and Miyaura made the initial discovery of rhodium-catalyzed addition to unsaturated systems, which yielded metal enolates in a safe and consistent manner. Amine 149 was treated with [Rh(COD)Cl], [PhB(OH)_2_], and KOH at 50 °C in dioxane/water solution (10 : 1). After 3 h, 150 was isolated with a yield of 21% of a mixture of 151 and 152, which was eliminated through Rh(i)-catalyzed Heck-type processes. Attempts to suppress these by-products using substrates such as 152 resulted in complex mixtures (Scheme 33). Although a crucial component of this strategy is the oxidative addition of an Rh(i) species into a C(aryl)X bond, there are fewer examples of this than for palladium and nickel.^112–119^

**Scheme 34:**
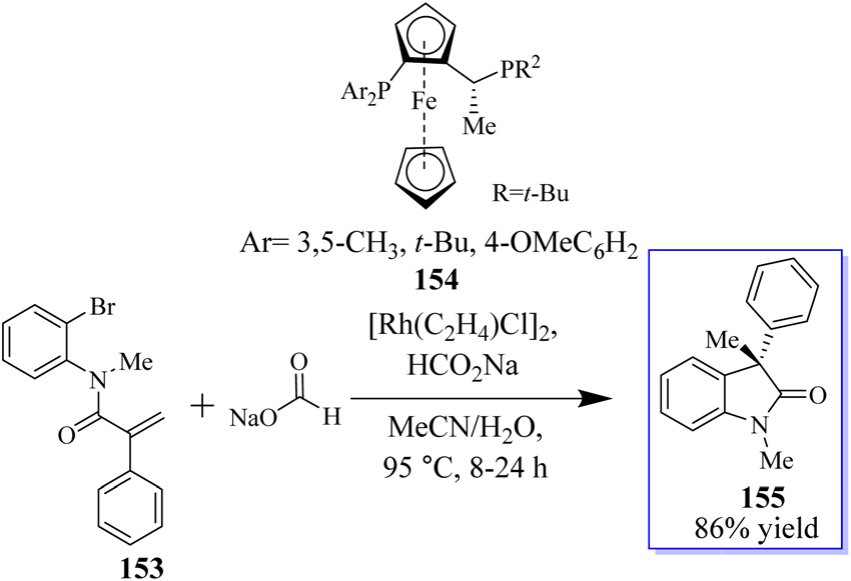
Reductive arylation induced by enantioselective Rh catalysis.

**Scheme 35:**
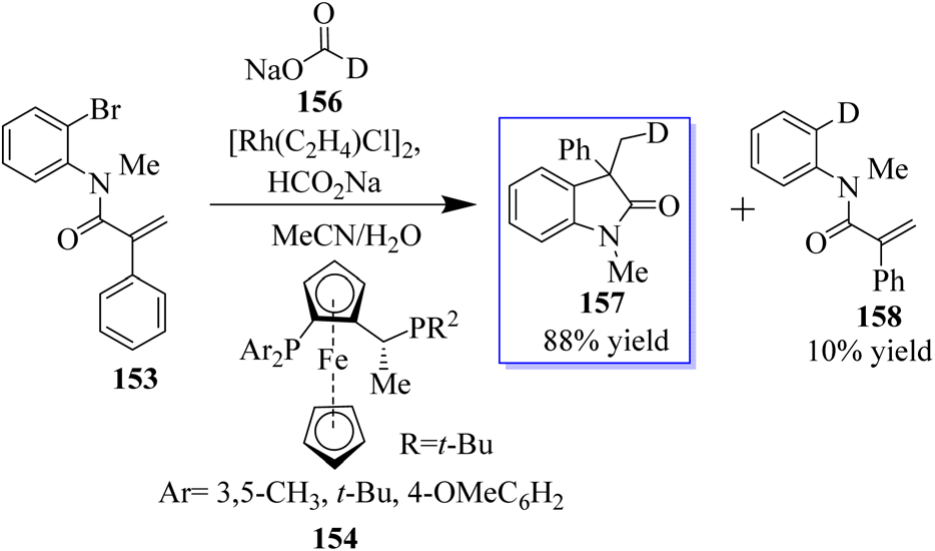
Enantioselective Rh-catalyzed reductive arylation.

The objective to make enantioselective chiral quaternary oxindole using ferrocene chiral ligands 154 was revealed. The investigation into other substitutes and nucleophiles was sparked by this result, specifically the addition of a hydride to acrylamide 153. The 3,3-disubstituted oxindoles 155 were delivered efficiently in up to 86% yield (Scheme 34).^120^ Conversely, 153 was subjected to the sodium deuterate format and H_2_O, making monodeuterated 157 the main product, which confirmed that the hydroxide actually came from sodium formate. In addition, small quantities of multi-deuterium and non-deuterium products were also observed in mass spectroscopy, possibly suggesting that deuterium incorporation can be a reversible process through the elimination of β-hydrides (Scheme 35).^120^

**Scheme 36:**
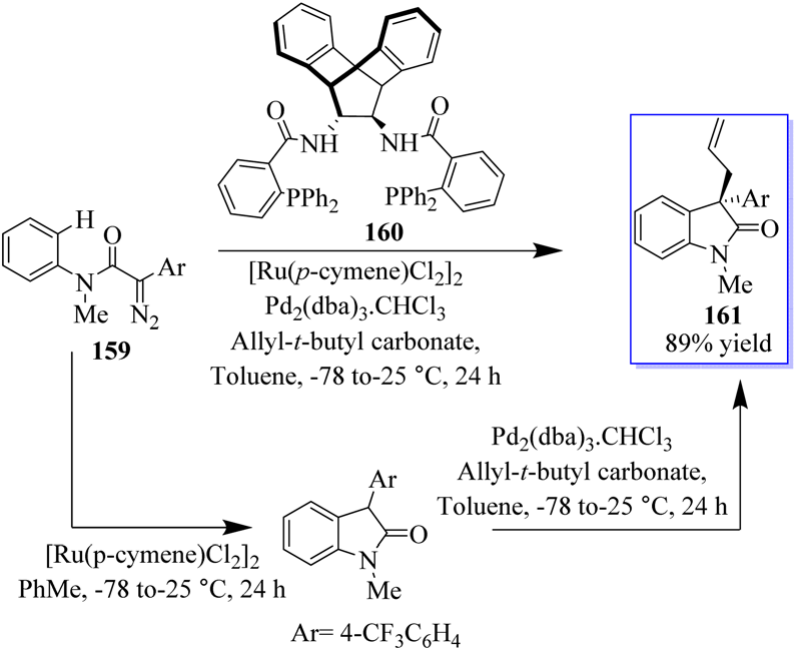
Enantioenriched 3,3-disubstituted oxindoles through a dual-metal approach.

Numerous groups have extensively researched the use of allylic electrophiles such as π-allylpalladium species, with the pioneering work by the Tsuji and Trost group. The authors optimized the transition metal-catalyzed C–H functionalization of α-diazoamide 159 substituting 4(trifluoromethyl)-phenyl-succinate to produce a high yield of enantioenriched oxindoles 161 based on Pd-catalyzed asymmetric allylic alkylation (AAA) using amine-based catalyst 160. In 2016, allyloxindoles were synthesized under the influence of Ru- and Pd-based catalysis (Scheme 36).^121–124^

**Scheme 37:**
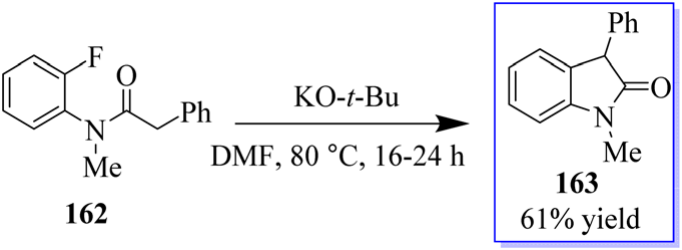
Synthesis of oxindole derivative by intramolecular α-arylation.

An interesting procedure to synthesize substituted oxindoles by intramolecular α-arylation of fluoro- and chloro-substituted anilides 162 in dimethylformamide at 80 °C, mediated by potassium *tert*-butoxide was revealed. Consequently, product 163 was produced in 61% yield (Scheme 37).^125^

**Scheme 38:**
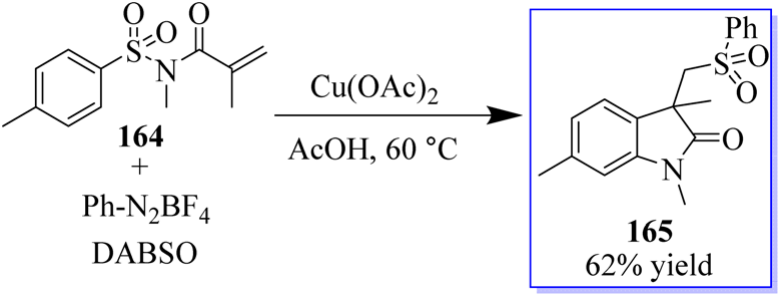
One-pot mechanism through radical addition reaction.

A three-component reaction involving *N*-(arylsulfonyl)acrylamides 164, DABSO (DABCO-bis(sulfur dioxide)), and phenyldiazonium tetrafluoroborates and Cu(OAc)_2_ was used to access sulfonated oxindoles 165. When aryldiazonium tetrafluoroborates and DABSO react, arylsulfonyl radicals are produced *in situ*, which initiates this transformation. Then, the formation of four new bonds occurs sequentially in one pot through radical addition, radical cyclization, and desulfonylative 1,4-aryl migration to produce the final product. This method exhibits high product yield and strong functional group tolerance (Scheme 38).^126^

**Scheme 39:**
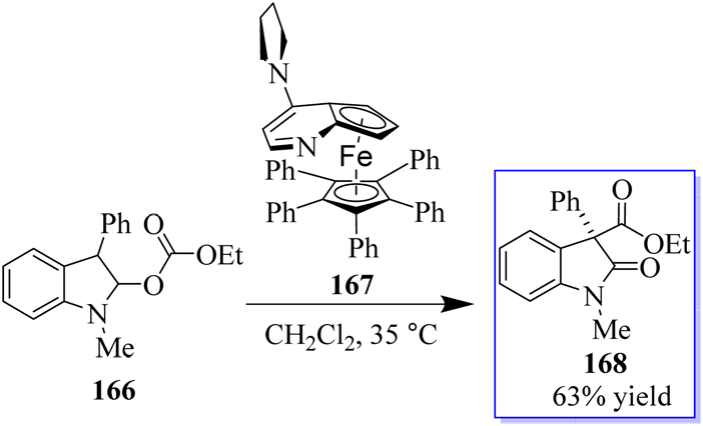
Effect of the acyl group on the O-to-C rearrangements.

**Scheme 40:**
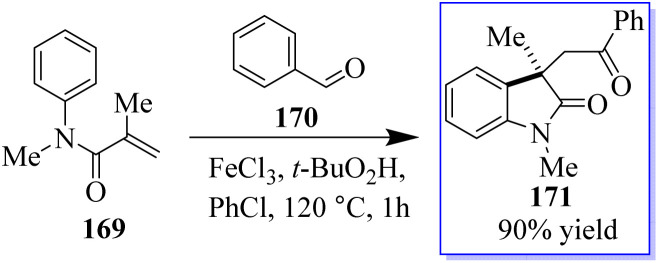
Oxidative carbonylation of alkenes with aldehydes.

The common natural product 168 derived from indole has a quaternary stereocenter at the C-3 position of heterocycle. Catalyst 167 promotes the asymmetric synthesis of these compounds by rearranging *O*-acylated oxindole 166 (Scheme 39).^127^ An effective method for the chiral synthesis of oxindole was established by carbonylation with aldehydes 170 and *N*-arylacrylamide 169. Three functionalized oxides 171 were smoothly synthesized in high yield with FeCl_3_ as the catalyst and tertiary butyl hydro-peroxide as the oxidizer. The obtained oxindoles can be used for further transformation to give various indole alkaloid structure motifs (Scheme 40).^128^

**Scheme 41:**
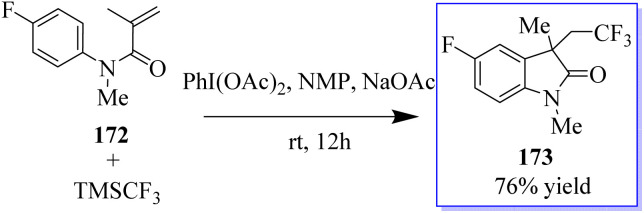
Main approaches to CF_3_-containing oxindoles.

Under metal-free conditions, a light and efficient trifluoromethylation of oxindole 173 was developed using *N*-aryl-acrylamide 172. This method is catalyzed by PhI(OAc)_2_ and mediated through TMSCF_3_. This method provides practical access to a variety of useful CF_3_-containing oxides with moderate to good yields (Scheme 41).^129^

**Scheme 42:**
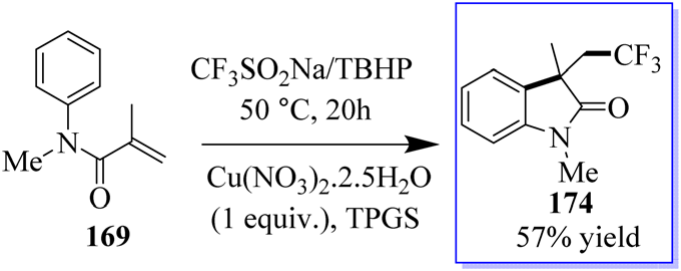
Transition-metal-catalyzed aryltrifluoromethylation of alkene.

The intramolecular trifluoromethylation of *N*-arylacrylamide 169 catalyzed by copper leads to oxide derivatives from the stable and cheap Langlois (CF_3_SO_2_Na) reagent to give corresponding oxindoles 174. These reactions occur through a radical process in water at ambient temperature. This method is advantageous in terms of being a green approach (Scheme 42).^130^

**Scheme 43:**
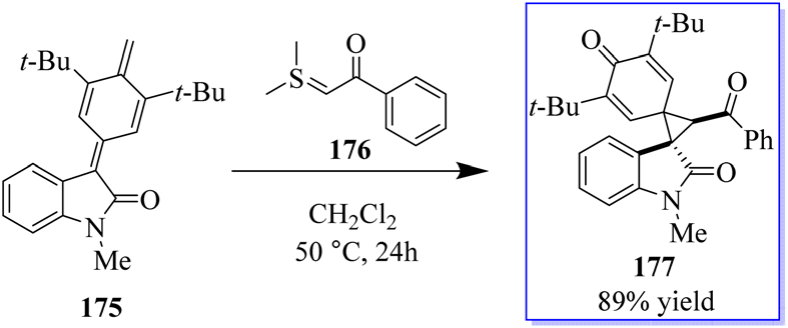
Applications of isatin-derived *p*-QMs for the synthesis of oxindole derivatives.

**Scheme 44:**
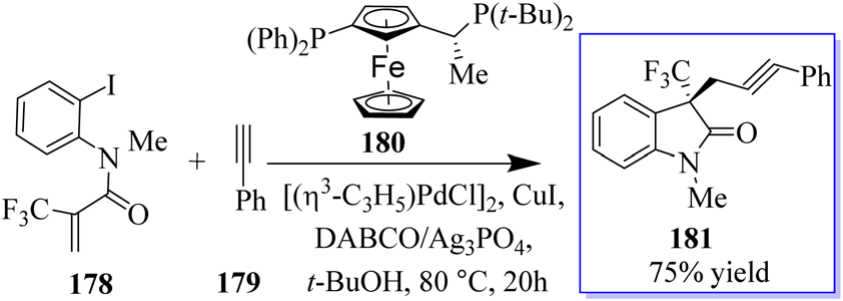
Enantioseletive synthesis of CF_3_-substituted oxindoles.

A catalyst-free and controllable reaction was developed between *para*-quinone methides derived from isatin 175 and sulfur ylides 176. This protocol allows the synthesis of different valuable oxindole derivatives 177 in a wide spectrum with high stereoselectivity (Scheme 43).^131^ An asymmetric palladium and copper sequential Heck/Sonogashira reaction between *o*-iodoacryl anilides and final alkyls was developed to synthesize chiral oxindoles 181 by the reaction of methyl 178 and *para*-methoxyphenyl (PMB) 179 in the presence of catalyst 180. A wide range of CF_3_-substituted *O*-iodoacryl anilides react with terminal alkynes and provide the corresponding chiral oxindoles with quadruple stereogenic trifluoromethylated centres in high isolated yields and excellent enantioselectivity. This asymmetric Heck/Sonogashira reaction provides a general approach for the insertion of oxindole derivatives containing quaternary stereogenic centers, including those that replace CF_3_ (Scheme 44).^132^

**Scheme 45:**
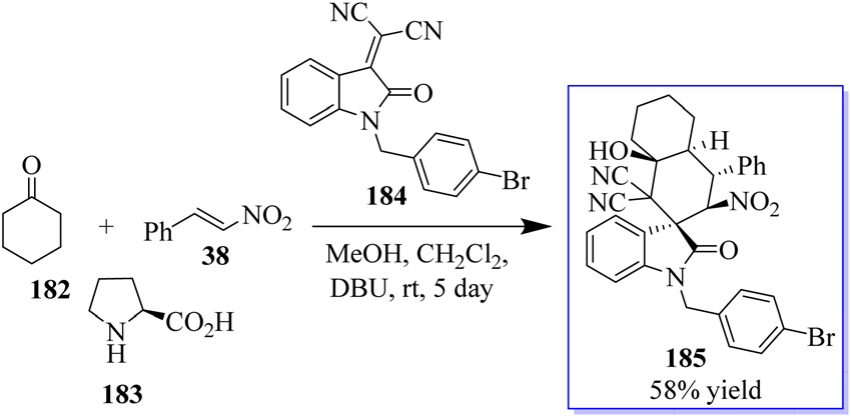
Synthesis of bioactive spirocyclic oxindoles.

The activation of cyclohexanone by enamine formation for cascade reactions is crucial for synthetic chemists. This concept was revealed using cyclohexanone 182, nitrostyrene 38, and l-proline 183. Subsequently, the first test reaction using substrate 184, in the presence of the basic organic catalyst DBU and MeOH to promote the domino Michael-aldol reaction was reported. The chiral oxindole having quaternary centre 185 was isolated in mild yield (Scheme 45).^133^

**Scheme 46:**
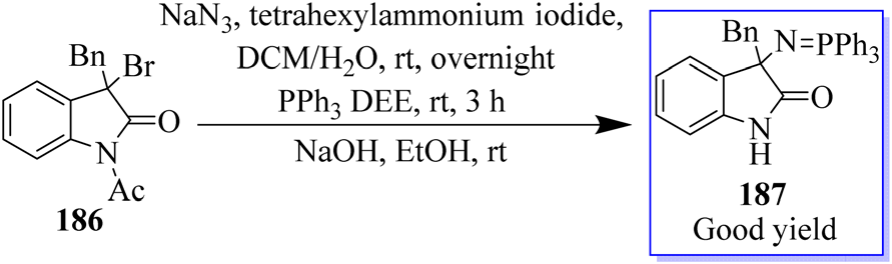
Synthesis of 3,3-disubstituted oxindole derivatives.

**Scheme 47:**
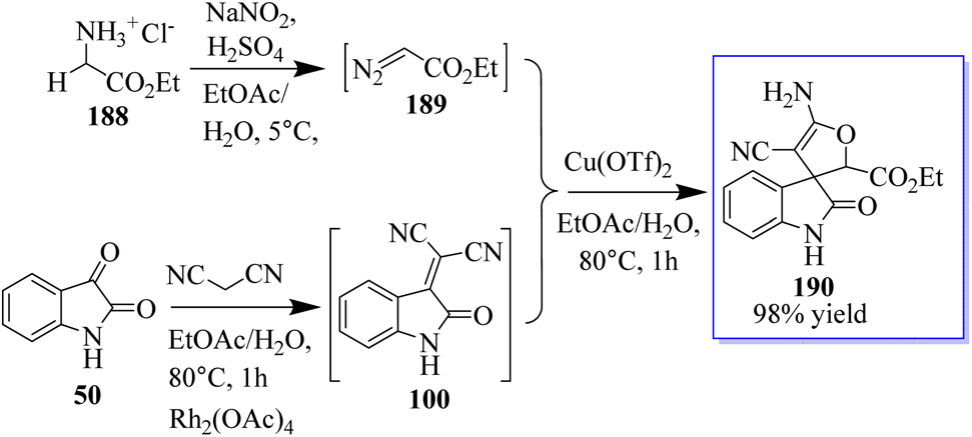
Multi-component cascade reactions.

Using 3-alkyloxindole 186, a derivative of oxindole was synthesized, beginning with the C-3 nitrogen atom. These derivatives were tested in five human tumor cell lines and in healthy donor primary cells (PBMCs), providing compounds with high anticancer effects in low micromolecular areas of all cancer cells. The authors reported the green synthesis of iminophosphorane-based oxindole derivatives 187, which are potentially useful in medicinal chemistry (Scheme 46).^134^ The Rh_2_(OAc)_4_-catalyzed multi-component reaction of *in situ*-generated ethyl diazoacetate (EDA) 189 and isatylidene malononitrile 100 in the two-phase solvent of water and ethyl acetate using copper triflate was described for the synthesis of chiral oxindole. The Michael-type adducts of hydroxyl oxonium ylide and malononitrile isatylidene underwent soft inner-molecular ring closure to obtain 190 as the final product with the excellent yield of 98% yield at 50 : 50 diastereoselectivity (Scheme 47).^135^

**Scheme 48:**
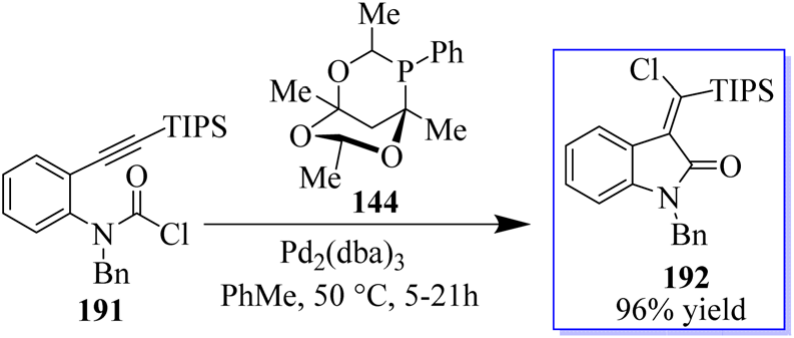
Carbamoyl chlorination reaction catalyzed by Pd(0).

The desired products 192 were only produced in very small amounts because this strategy was incompatible with the synthesis of methylene oxindoles. Using carbamoyl chloride 191 tethered to an alkyne moiety, a different approach that may provide access to the highly desired halogenated methylene oxindole scaffold was discovered. The reaction, which made use of Pd_2_(dba)_3_ and the large phosphaadamantane ligand 144, proved to be extremely selective by directing the reductive elimination *trans* to the initial carbopalladation site (Scheme 48).^136^

**Scheme 49:**
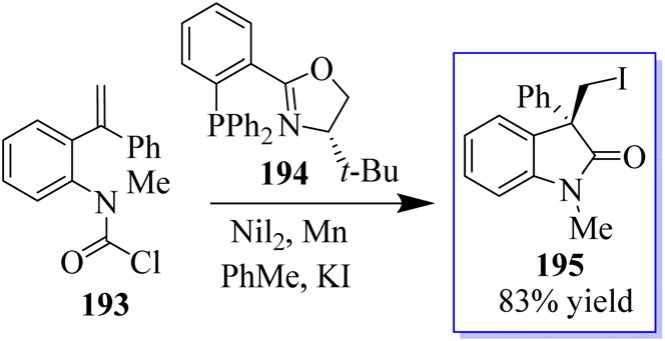
Carbamoyl iodination process catalyzed by enantiospecific nickel.

Due to inability of nickel to form a C(sp^3^)–Cl bond, an external iodide source had to be added to carry out in-place halogen exchange and enable the reductive elimination of the C(sp^3^)–I bond. The nickel-catalyzed cross-coupling cyclization reactions were carried out with carbamoyl chlorides 193 as the electrophiles. The asymmetric transfer of a carbamoyl chloride surrogate across a tethered disubstituted styrene using Ni-*t*-BuPHOX catalyst in the presence of Mn(0) and a nucleophilic source of iodide (KI) produced oxindole derivatives 195 having a chlorine moiety (Scheme 49).^137^

**Scheme 50:**
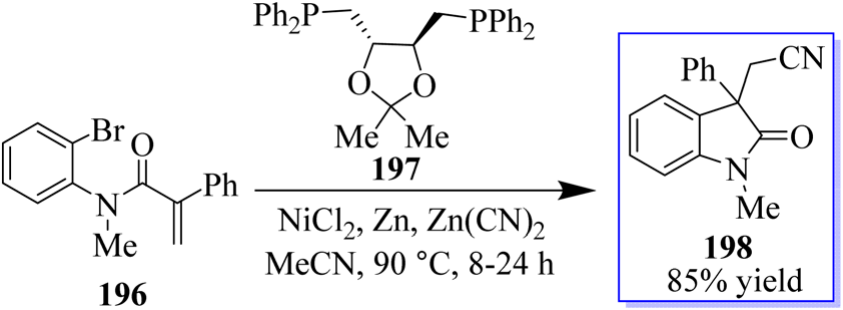
Reaction of arylcyanation catalyzed by nickel.

The authors reported that Zn(CN)_2_ was used as the source of cyanide in the NiCl_2_(glyme)DIOP catalytic system, which produced cyanated oxindole products (Scheme 50). By adding activated zinc dust to the reaction mixture, the air-sensitive Ni(COD)_2_ was avoided, enabling the use of the manageable (S,S)-DIOP 197 as the catalyst. The “chain walking” reaction, which was popularized by the work of Martin and Marek and others, produced a remotely cyanated oxindole 198 scaffold in one substrate.^138,139^

## 8. Conclusions

In this account, we discuss the outcomes of the design, development, and use of synthetic methodologies for the catalytic enantioselective synthesis of 3,3-disubstituted oxindoles. We reported several effective protocols based on different techniques that are categorized by the oxindole synthons used, making it simple to access oxindole-based natural products and synthetic derivatives with a wide range of structural diversity. Also, we reported the synthetic methodologies with a variety of potential bioactive applications. The development of quinine, thioquinine, and phosphoramide-based chiral bifunctional catalysts or ligands is important given that the success of bifunctional catalysts in the Michael addition, which have some advantages over other H-bond donors. Additionally, the chemical reactivity of 2-oxindole-based derivatives was also described to understand the chemical reactivity pattern of this class of molecules. The use of Mizoroki–Heck-inspired domino cyclization reactions, 1,2-addition–cyclization domino sequences, and MCR strategy involving C–H functionalization can produce and derivatize these valuable structures. Although all these methods build the basic oxindole scaffold, each one has advantages and depends on a wide variety of fundamental reactivity. We anticipate that the techniques and approaches created will serve as models for developing approaches for other biologically significant scaffolds.

## Author contributions

Shivangi Sharma: writing original draft, software. Yukti Monga: formal analysis. Ashu Gupta: formal analysis. Shivendra Singh: writing, review and editing.

## Conflicts of interest

There are no conflicts of interest to declare.

## Supplementary Material
